# Molecular Characteristics, Potential Mechanisms, and Prognostic Gene Model of Younger Female Patients With Gastric Cancer

**DOI:** 10.1002/cnr2.70469

**Published:** 2026-03-03

**Authors:** Xiaoyi Luan, Lulu Zhao, Wanqing Wang, Penghui Niu, Xue Han, Zerong Wang, Xiaojie Zhang, Dongbing Zhao, Yingtai Chen

**Affiliations:** ^1^ National Cancer Center/National Clinical Research Center for Cancer/Cancer Hospital Chinese Academy of Medical Sciences and Peking Union Medical College Beijing China

**Keywords:** gastric cancer, pathogenesis, prognostic gene model, sex

## Abstract

**Background:**

Male patients were twice as likely to develop gastric cancer (GC) compared to females, partly due to the protective effect of estrogen. However, the proportion of females increased in the young GC patients.

**Aims:**

The study was designed to explore comprehensive molecular profiles of younger female GC patients, as well as develop a prognostic gene model for female GC patients.

**Materials&Methods:**

Gene expression and clinical data of GC and nontumor patients were downloaded from the Gene Expression Omnibus (GEO) database. Gene Ontology (GO), Kyoto Encyclopedia of Genes and Genomes (KEGG), and gene set enrichment analysis (GSEA) were used to find molecular characteristics and potential mechanisms of younger female GC patients. The prognostic gene model containing six differential expressed genes (DEGs), which were between younger and older female patients, was established using Lasso–Cox regression. Its performance was validated by external validation. Then, receiver operating characteristic (ROC) curve was applied to determine the prognostic value of the prognostic gene model.

**Results:**

Six GEO cohorts with 305 female GC patients (69 younger patients and 236 older patients) and 38 female nontumor patients were included. A total of 4557 DEGs between female GC patients and nontumor patients were identified, including 2212 upregulated genes and 2345 downregulated genes. Estrogen response early (*p* < 0.001) and estrogen response late (*p* < 0.001) were enriched in female GC patients. In KEGG analysis, aldosterone (*p* = 0.023) and relaxin pathways (*p* = 0.043) were concentrated in the younger group. Moreover, we further used the GSE84437 cohort to construct a prognostic gene model containing six genes, namely NREP, GAD1, SLCO4A1, KRT17, DEFB1, and P3H2, to predict the overall survival (OS) of female GC patients (AUC = 0.810). In the overall analysis of female GC patients, high‐risk patients showed worse OS than low‐risk patients (HR = 5.7688, 95% CI: 3.0108–11.0530, *p* < 0.001). Compared with older female patients, younger female patients had a higher tendency to be in the high‐risk group (31.1% vs. 18.3%, *p* = 0.018).

**Conclusions:**

In conclusion, we provided the comprehensive molecular profiles of younger female GC patients and found that there was a significant difference in enriched hormone‐related pathways between the younger group and the older group. Compared with older female patients, younger female patients were more likely to be in the high‐risk group, which showed worse OS than low‐risk patients.

## 1 Introduction

Gastric cancer (GC) is the fifth most common cancer and the fourth leading cause of cancer‐related death worldwide [1, 2]. Male patients were twice as likely to develop GC compared to females [3]. However, the proportion of females increased in the young GC patients, with the female/male ratio ranging from 1:1 to 2:1 [4, 5, 6, 7, 8, 9, 10, 11]. Although several studies believed the female dominance in the young GC patients was related to hormonal factors, the basis of this marked disparity in incidence was poorly understood [6, 12].

In this context, an increasing number of researchers have conducted mechanism studies in the field of younger patients with GC [13, 14, 15, 16, 17, 18]. Previous studies suggested that the germline mutation of the CDH1 gene might lead to hereditary diffuse GC in younger patients [13, 14, 15, 16, 17], however, the conclusions above did not focus on younger female patients only. Only one study, on the basis of cell lines, found that estrogen‐induced transcription of HOX antisense intergenic RNA (HOTAIR) might contribute to the pathogenesis mechanisms in diffuse GC of younger female patients [18]. However, this finding only focused on the vitro grown cell lines supported by an artificial niche, which might not closely model the in vivo environment. Therefore, the mechanism lacked sufficiently convincing evidence from animal models or population cohorts [19]. In addition, it has been gradually acknowledged that younger female GC patients had a poorer prognosis compared to the older with an abundance of clinical evidence [4, 20, 21, 22, 23, 24]. However, little was known concerning the genetic mechanisms of poor prognosis in younger female patients with GC.

As such, our study aimed to explore the molecular characteristics and potential mechanisms of younger female GC patients and also to construct a prognostic gene model for evaluating the prognosis of female patients with GC, which provided valuable biological insights into this disease.

## 2 Methods

### 2.1 Data Collection and Sample Information

**FIGURE 1:**
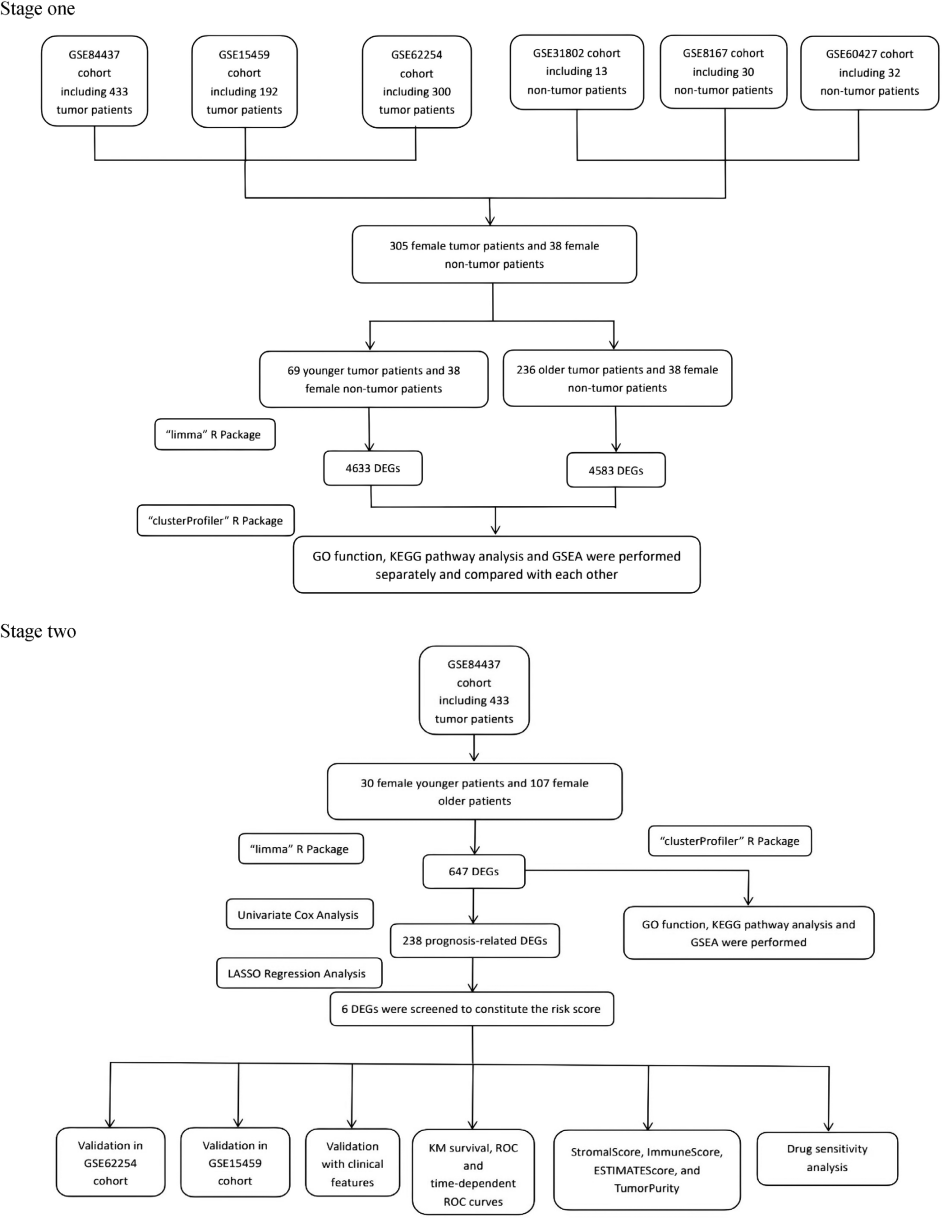
The overall analysis of this study.

The overall design of this study is shown in Figure 1. Gene expression and clinical data of GC and nontumor patients were downloaded from the GEO database. The main inclusion criteria for the GC cohort were: (I) primary lesion diagnosed as GC; (II) complete information on gender, age, and survival; and (III) availability of RNA transcriptomic data in the cohort. The exclusion criteria were: (I) patients with other diseases or cancers that affected hormone levels; (II) incomplete information on gender, age, or survival; (III) special‐type GC; (IV) excessive disparity in the number of young and elderly female patients in the cohort; and (V) dual‐channel microarray. After screening, the only suitable GC cohorts were GSE84437, GSE15459, and GSE62254. The nontumor group, including 38 female nontumor patients, was provided by three other cohorts (GSE31802, GSE8167, and GSE60427). They underwent gastrectomy due to benign gastric diseases, such as gastrointestinal stromal tumors. The discovery cohort contained 137 female GC patients retrieved from the GEO database (GSE84437), which was used in the construction of the prognostic gene model. Two independent cohorts were used for external validations (GSE15459 and GSE62254). In addition, 137 female GC samples in South Korea (GSE84437), 67 female GC samples in Singapore (GSE15459), and 101 GC samples in the Asian Cancer Research Group (ACRG) study (GSE62254) had complete characteristic information and survival duration, which were available at: https://www.ncbi.nlm.nih.gov/geo/. Batch effects were removed with the ComBat function of the “sva” package. All datasets were normalized using “limma” package.

### 2.2 Identification of DECs in GC

All datasets were downloaded through the “GEOquery” package. The “limma” package was used to screen DEGs. The criteria for DEGs between GC patients and nontumor controls was |log_2_FC| > 1 and *p* < 0.05 (FC, fold change). The DEGs between younger and older female GC patients were defined as |log_2_FC| > 0.5 and *p* < 0.05.

### 2.3 Functional and Pathway Enrichment Analysis

Functional annotation of the DEGs was implemented via “clusterProfiler” package, and the further analysis of Gene Ontology (GO) and Kyoto Encyclopedia of Genes and Genomes (KEGG) was performed. The gene set enrichment analysis (GSEA) was also conducted for ascertaining the difference in pathways through “clusterProfiler” package. The gene sets of “c5.all.v7.0.entrez” and “h.all.v7.0.entrez” were acquired from the Molecular Signatures Database (MSigDB) to run GSEA. A pathway term with |Normalized Enrichment Score (NES)| > 1, adjusted *p* < 0.05, and false discovery rate (FDR) < 0.25 was considered to be significantly enriched. A bar chart was plotted by https://www.bioinformatics.com.cn (last accessed on July 10, 2023), an online platform for data analysis and visualization.

### 2.4 Construction and Validation of a Prognostic Risk Model

The 266 candidate DEGs related to prognosis were selected through “survival” package. Survival‐related DEGs were subjected to Lasso Cox regression analysis, and six genes were finally identified and involved in the construction of the prognostic gene model, including NREP, GAD1, SLCO4A1, KRT17, DEFB1, and P3H2. The risk score was calculated according to the following formula: risk score = $\sum_{i=1}^{n}\left(\text{Expi}\times \text{Coefi}\right)$.

*Expi* represents the gene expression levels, and *Coefi* represents the risk coefficients. The cutoff point of risk score was determined via “survminer” package. Based on the cutoff point of discovery cohort, patients from discovery cohort were divided into low‐risk and high‐risk groups. The ratio of high‐risk/low‐risk in the discovery cohort was used as the criteria for dividing the external validation cohort.

### 2.5 Construction and Validation of the Prognostic Gene Model

The differences in overall survival (OS) between low‐risk group and high‐risk group were evaluated through the Kaplan–Meier analysis generated by “survival” package. For the predicted assessment of female patients with GC in the prognostic value of the prognostic gene model, the receiver operating characteristic (ROC) curve analysis and the time‐dependent ROC curve analysis were performed for obtaining the area under the curve (AUC). We performed univariate and multivariate Cox proportional hazards regression for all cohorts to determine whether the risk score was an independent predictor of prognosis.

### 2.6 Assessment of Tumor Microenvironment (TME)

In order to explore the changes of TME in female GC patients between different risks, we used “estimate” package to score the stromal score, immune score, ESTMATE score, and tumor purity of three cohorts through the algorithm.

### 2.7 Drug Sensitivity Analysis

In order to study the clinical manifestations of chemotherapy drugs in female patients, we calculated the semi‐inhibitory concentration (IC50) values of 198 drugs through “oncoPredict” package. Wilcoxon signed‐rank test was used to explore the difference in IC50 between low‐risk and high‐risk groups. The results were performed via “ggpubr” package.

### 2.8 Statistical Analysis

The correlations between continuous variables were assessed using Spearman correlation analysis, and differences in the variables between different risk groups were evaluated with the Student *t*‐test, one‐way ANOVA, Pearson's chi‐squared test, or Wilcoxon signed‐rank test. Results with two‐sided *p* < 0.05 were deemed significant. The statistical analysis of this study was performed using R‐4.3.0 software (2023‐04‐21 ucrt).

## 3 Results

### 3.1 Characteristics of Females GC Patients

The clinicopathologic characteristics of female GC patients were listed in Table 1. There was no statistically significant difference in OS between younger and older female patients (*p* = 0.85). In comparison to older female patients, younger female patients were more likely to be Lauren diffuse‐type GC (84.6% vs. 53.5%, *p* = 0.002). No variations in tumor stage were observed between the two groups (*p* > 0.05).

**TABLE 1:** The clinicopathologic characteristics of female GC patients.

Characteristics	Overall (*N* = 305)		Younger (*N* = 69)		Older (*N* = 236)		*p*
	*N*, median	%, [IQR]	*N*, median	%, [IQR]	*N*, median	%, [IQR]	
Age (years)	63	[52.00, 70.00]	42	[36.00, 47.00]	66.1	[59.00, 72.08]	< 0.001
Group							
Younger	69	22.60%	—	—	—	—	—
Older	236	77.40%	—	—	—	—	—
OS (days)	1740	[450.90, 2778.90]	1355.1	[570.00, 2739.00]	1778.55	[424.50, 2780.70]	0.85
Lauren							
Intestinal	61	36.30%	6	15.40%	55	42.60%	0.002
Diffuse	102	60.70%	33	84.60%	69	53.50%	
Mixed	5	3.00%	0	0.00%	5	3.90%	
*T*							
1	4	1.70%	0	0.00%	4	2.20%	0.504
2	68	28.60%	12	23.10%	56	30.10%	
3	60	25.20%	14	26.90%	46	24.70%	
4	106	44.50%	26	50.00%	80	43.00%	
*N*							
0	41	17.20%	8	15.40%	33	17.70%	0.83
1	107	45.00%	24	46.20%	83	44.60%	
2	61	25.60%	12	23.10%	49	26.30%	
3	29	12.20%	8	15.40%	21	11.30%	
*M*							
0	87	86.10%	17	77.30%	70	88.60%	0.312
1	14	13.90%	5	22.70%	9	11.40%	
pStage							
1	25	14.90%	4	10.30%	21	16.30%	0.196
2	43	25.60%	6	15.40%	37	28.70%	
3	49	29.20%	14	35.90%	35	27.10%	
4	51	30.40%	15	38.50%	36	27.90%	

**FIGURE 2:**
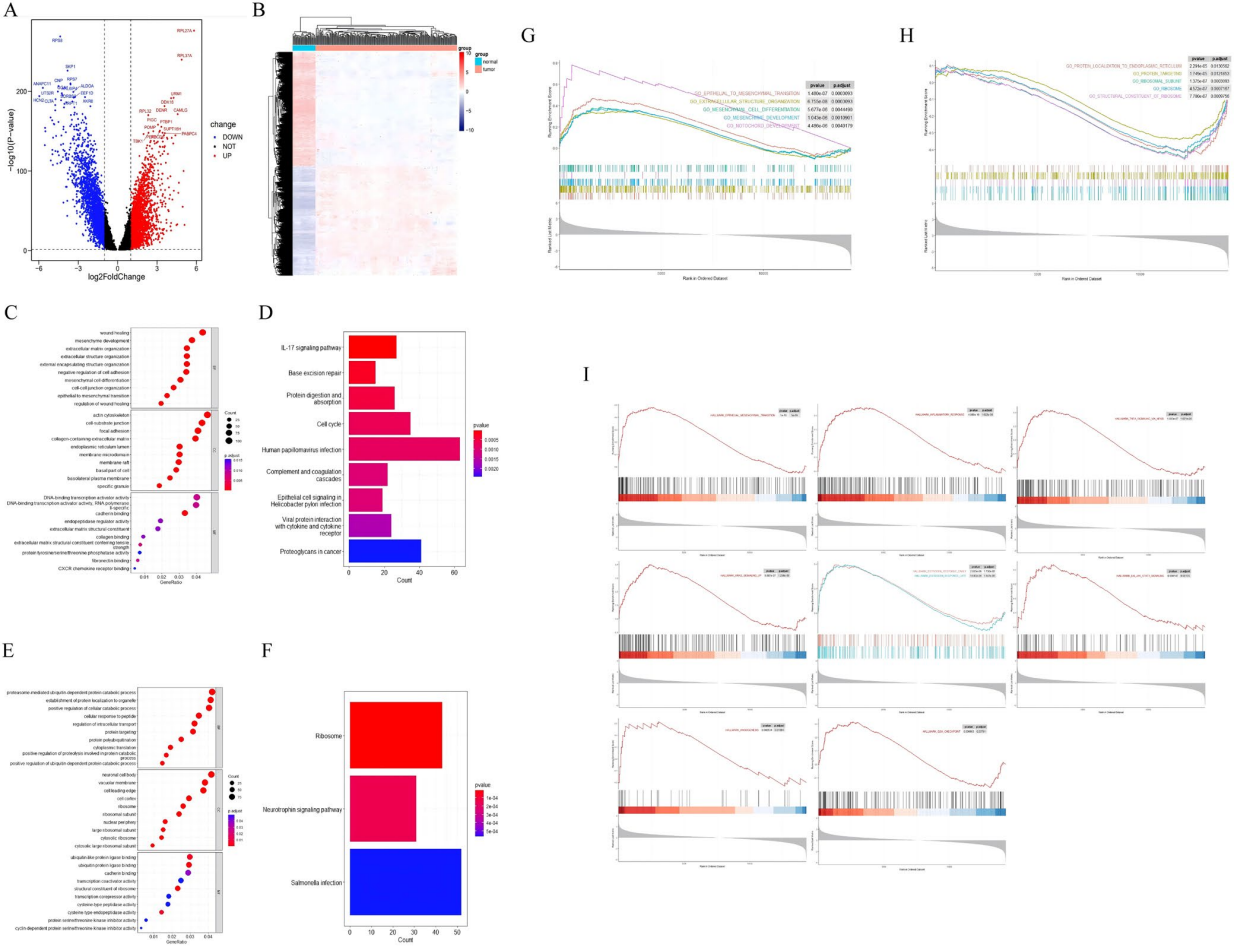
The DEGs between female gastric cancer (GC) patients and nontumor controls. (A) Volcano plot of the DEGs with the top 15 DEGs. (B) Heat map of the DEGs. (C and D) GO function, KEGG pathway analysis of upregulated DEGs. (E and F) GO function, KEGG pathway analysis of downregulated DEGs. (G) Enrichment plots from GSEA in C5 collection of upregulated genes (The red curve represented the “GO_EPITHELIAL_TO_MESENCHYMAL_TRANSITION” pathway, *p* = 1.480e−07, *p*.adjust = 0.0003093. The green curve represented the “GO_EXTRACELLULAR_STRUCTURE_ORGANIZATION” pathway, *p* = 6.755e−08, *p*.adjust = 0.0003093. The light‐blue curve represented the “GO_MESENCHYMAL_CELL_DIFFERENTIATION” pathway, *p* = 5.677e−06, *p*.adjust = 0.0044490. The dark‐blue curve represented the “GO_MESENCHYME_DEVELOPMENT” pathway, *p* = 1.043e−06, *p*.adjust = 0.0010901. The purple curve represented the “GO_NOTOCHORD_DEVELOPMENT” pathway, *p* = 4.486e−06, *p*.adjust = 0.0040179). (H) Enrichment plots from GSEA in C5 collection of downregulated genes (The red curve represented the “GO_PROTEIN_LOCALIZATION_TO_ENDOPLASMIC_RETICULUM” pathway, *p* = 2.291e−05, *p*.adjust = 0.0130562. The green curve represented the “GO_PROTEIN_TARGETING” pathway, *p* = 1.749e−05, *p*.adjust = 0.0121853. The light‐blue curve represented the “GO_RIBOSOMAL_SUBUNIT” pathway, *p* = 1.375e−07, *p*.adjust = 0.0003093. The dark‐blue curve represented the “GO_RIBOSOME” pathway, *p* = 4.572e−07, *p*.adjust = 0.0007167. The purple curve represented the “GO_STRUCTURAL_CONSTITUENT_OF_RIBOSOME” pathway, *p* = 7.780e−07, *p*.adjust = 0.0009756). (I) Pathways associated with tumor progression in female GC patients.

To investigate the molecular characteristics of female GC patients, a total of 4557 DEGs, including 2212 upregulated genes and 2345 downregulated genes, were identified, which were statistically significant between female GC patients and nontumor controls (Figure 2A). The top five upregulated genes were RPL27A, CDH17, COL6A3, GCNT3, and RPL37A. The specific expression of the screened DEGs was demonstrated by the heatmap as shown in Figure 2B.

In order to further explore the underlying mechanisms involved in female GC development, functional enrichment analyses of the DEGs were conducted. GO analysis indicated that in biological processes (BP), upregulated genes were mainly enriched in wound healing, mesenchyme development, extracellular matrix organization, extracellular structure organization, external encapsulating structure organization, and downregulated genes were mainly enriched in proteasome‐mediated ubiquitin‐dependent protein catabolic process, establishment of protein localization to organelle, positive regulation of cellular catabolic process, cellular response to peptide, regulation of intracellular transport. The cell component (CC) and molecular function (MF) of GO analysis were shown in Figure 2C,E. Moreover, the KEGG analysis revealed that upregulated genes were enriched in IL‐17 signaling pathway, base excision repair, protein digestion and absorption, cell cycle, human papillomavirus (HPV) infection, epithelial cell signaling in Hp infection, and downregulated genes were enriched in ribosome, neurotrophin signaling pathway, salmonella infection (Figure 2D,F). Combined with the results of GO and KEGG, we found that the DEGs between female GC patients and nontumor controls were significantly enriched in metabolic pathways, extracellular structure, cell cycle, and Hp infection.

Enrichment plots from GSEA in C5 collection were shown in Figure 2G,F. The results of GSEA based on the Hallmarker gene sets showed that epithelial–mesenchymal transition (EMT), inflammatory response, TNFα signaling via NF‐κB, KRAS signaling, estrogen response early and late, IL‐6/JAK/STAT3 signaling, angiogenesis, and G2M checkpoint were significantly upregulated in female GC patients, which suggested there was a potential relationship between GC and estrogen pathways (estrogen response early: NES = 1.838, *p* < 0.001, FDR < 0.001; estrogen response late: NES = 1.842, *p* < 0.001, FDR < 0.001, Figure 2I).

### 3.2 Differences in Potential Mechanisms Between Younger and Older Female GC Patients

**FIGURE 3:**
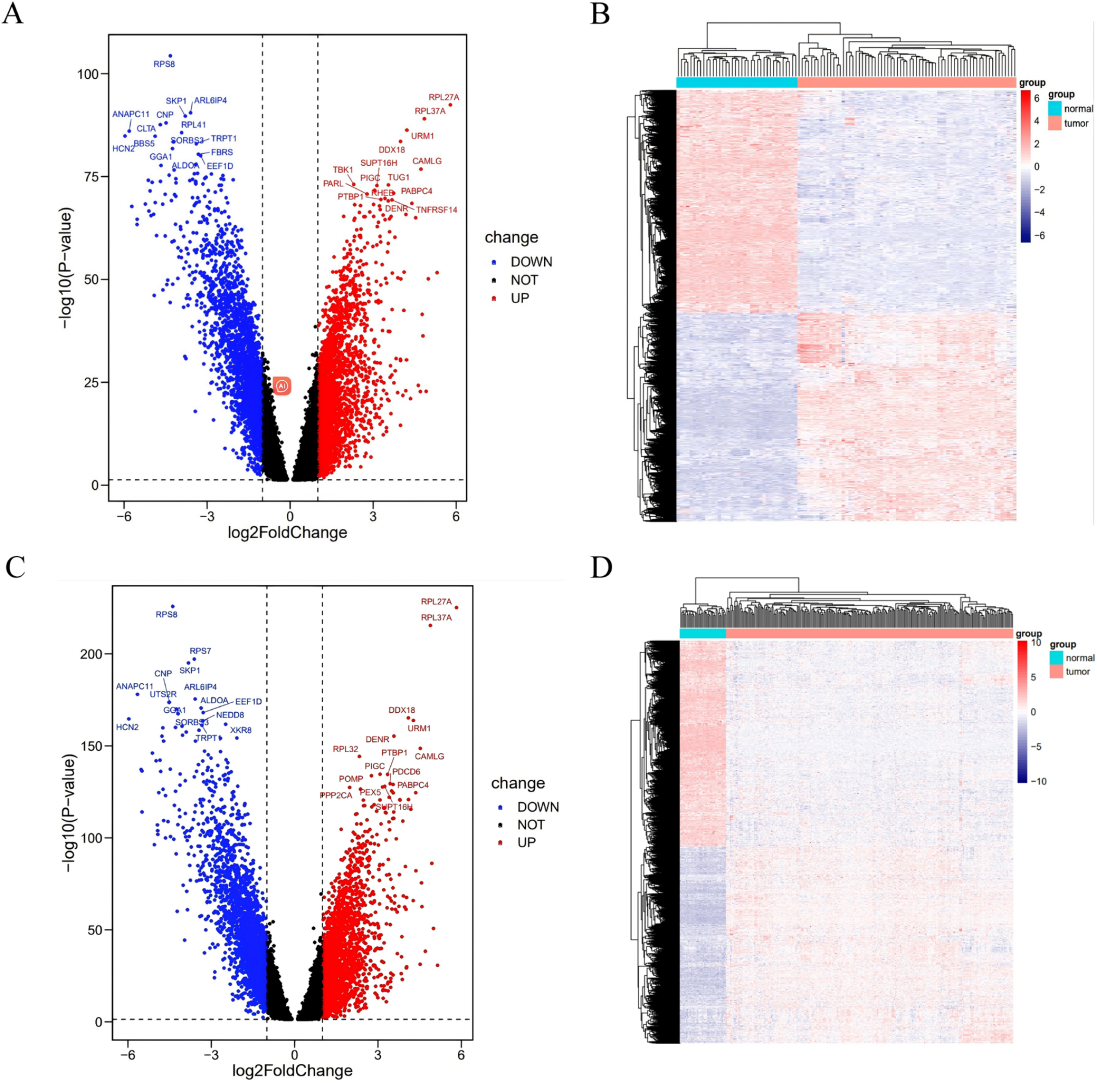
The differentially expressed genes (DEGs) of the two groups. (A) Volcano plot of the DEGs with the top 15 DEGs in the younger group. (B) Heat map of the DEGs in the younger group. (C) Volcano plot of the DEGs with the top 15 DEGs in the older group. (D) Heat map of the DEGs in the older group.

Female GC patients included in our study were divided into two groups according to age 50 (≤ 50 years of age, *n* = 69; > 50 years of age, *n* = 236). A total of 4633 DEGs were identified in the younger group, including 2241 upregulated genes and 2392 downregulated genes. In the older group, a total of 4583 DEGs were identified, including 2241 upregulated genes and 2342 downregulated genes. The DEGs of the two groups were displayed by volcano maps as shown in Figure 3A,C. The distribution of the DEGs was demonstrated by the heatmaps as shown in Figure 3B,D.

**FIGURE 4:**
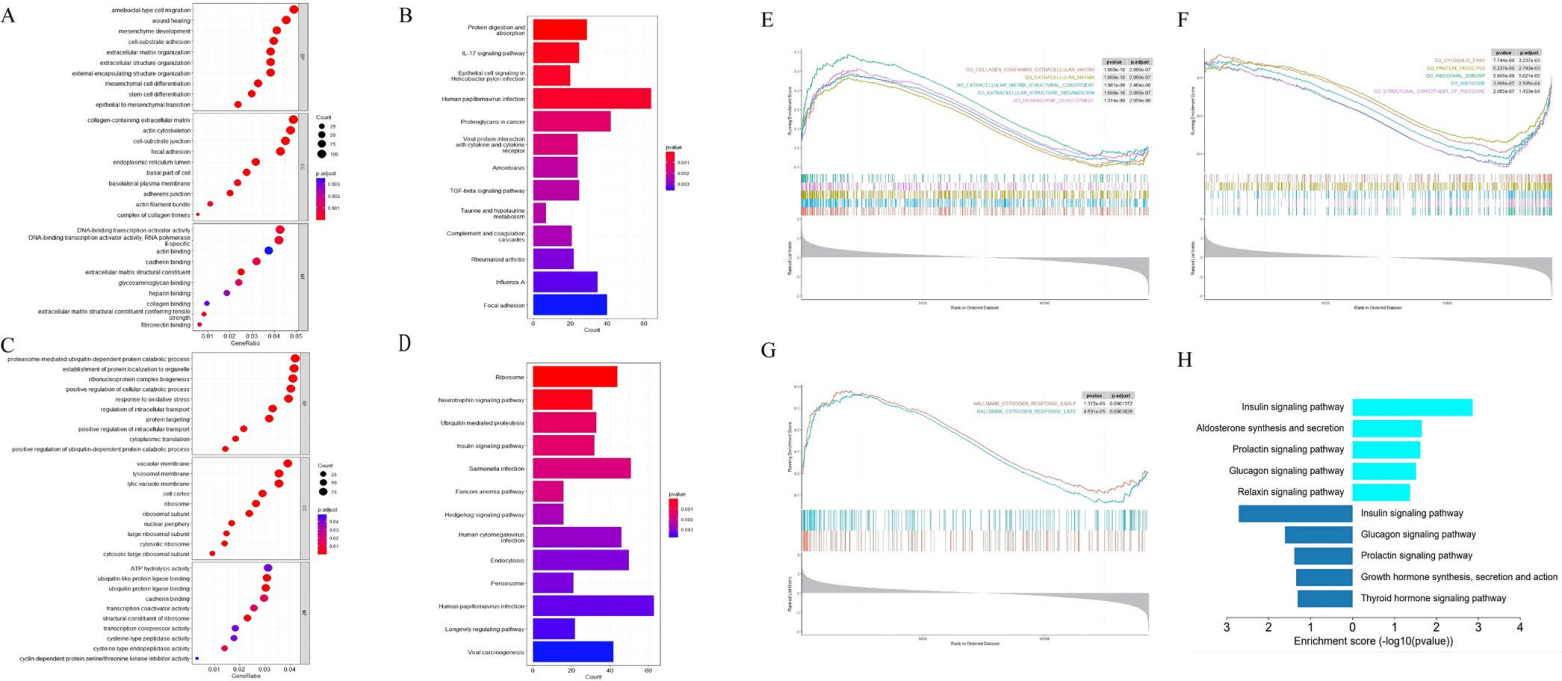
Functional annotation of DEGs in female GC patients of the younger group. (A and B) GO function, KEGG pathway analysis of upregulated DEGs. (C and D) GO function, KEGG pathway analysis of downregulated DEGs. (E) Enrichment plots from GSEA in C5 collection of upregulated genes (The red curve represented the “GO COLLAGEN CONTAINING EXTRACELLULAR MATRIX” pathway, *p* = 1.000e−10, *p*.adjust = 2.090e−07. The green curve represented the “GO EXTRACELLULAR MATRIX” pathway, *p* = 1.000e−10, *p*.adjust = 2.090e−07. The light‐blue curve represented the “GO EXTRACELLULAR MATRIX STRUCTURAL CONSTITUENT” pathway, *p* = 1.981e−09, *p*.adjust = 2.484e−06. The dark‐blue curve represented the “GO EXTRACELLULAR STRUCTURE ORGANIZATION” pathway, *p* = 1.000e−10, *p*.adjust = 2.090e−07. The purple curve represented the “GO MESENCHYME DEVELOPMENT” pathway, *p* = 1.314e−09, *p*.adjust = 2.059e−06). (F) Enrichment plots from GSEA in C5 collection of downregulated genes (The red curve represented the “GO_CYTOSOLIC_PART” pathway, *p* = 7.744e−06, *p*.adjust = 3.237e−03. The green curve represented the “GO_PROTEIN_TARGETING” pathway, *p* = 6.237e−06, *p*.adjust = 2.793e−03. The light‐blue curve represented the “GO_RIBOSOMAL_SUBUNIT” pathway, *p* = 5.605e−08, *p*.adjust = 5.021e−05. The dark‐blue curve represented the “GO_RIBOSOME” pathway, *p* = 3.995e−07, *p*.adjust = 2.505e−04. The purple curve represented the “GO_STRUCTURAL_CONSTITUENT_OF_RIBOSOME” pathway, *p* = 2.085e−07, *p*.adjust = 1.453e−04). (G) Endocrine‐related pathways in the enrichment plot from GSEA in HALLMARK collection. (H) Differences in endocrine‐related pathways in KEGG pathway analysis between the younger and older groups.

In order to further explore the potential mechanisms involved in GC development of younger female patients, the enrichment analysis of GO and KEGG were performed on 4633 DEGs between younger female patients and nontumor controls. Enrichment results of GO analysis were shown in Figure 4A,C. GO analysis indicated that in BP, upregulated genes were mainly enriched in ameboidal‐type cell migration, wound healing, mesenchyme development, cell‐substrate adhesion, extracellular matrix organization, extracellular structure organization, and downregulated genes were mainly enriched in proteasome‐mediated ubiquitin‐dependent protein catabolic process, establishment of protein localization to organelle, ribonucleoprotein complex biogenesis, positive regulation of cellular catabolic process, response to oxidative stress. The KEGG pathways related to upregulated genes were mainly concentrated in the pathways of protein digestion and absorption, IL‐17 signaling pathway, epithelial cell signaling in Hp infection, HPV infection, proteoglycans in cancer. Considering the downregulated genes, the KEGG pathways were mainly concentrated in the pathways of ribosome, neurotrophin signaling pathway, ubiquitin‐mediated proteolysis, insulin signaling pathway, salmonella infection. As the results of GSEA based on the Hallmarker gene sets, estrogen response early and late were enriched in younger group (estrogen response early: NES = 1.774, *p* < 0.001, FDR < 0.001; estrogen response late: NES = 1.699, *p* < 0.001, FDR < 0.001, Figure 4G). Other results were shown in Figure 4.

Functional and pathway enrichment analysis of the DEGs of older group was shown in Figure S1. Combining the results of the two groups, there was no significant difference in enriched pathways between younger group and older group in GO analysis. The above GO terms were involved in cancer occurrence and development to some extent, especially the upregulated DEGs which were majorly associated with metabolic pathways and extracellular structure (Figure S1A). Comparing the KEGG analysis results of the two groups, we found GC in the two groups were both related to Hp infection, because the DEGs were enriched in IL‐17 signaling pathway and epithelial cell signaling in Hp infection. However, there was a significant difference in enriched hormone‐related pathways between the two groups. The downregulated genes of the two groups were both enriched in insulin, prolactin, and glucagon signaling pathway. Aldosterone (*p* = 0.023) and relaxin pathways (*p* = 0.043) were concentrated in younger group, while growth hormone synthesis, secretion, and action (*p* = 0.046) and thyroid hormone signaling pathway (*p* = 0.049) were concentrated in older group (Figure 4H). The results of GSEA based on the Hallmarker gene sets showed that estrogen response early and late were enriched in older group, which were consistent with the results of GSEA in younger group (estrogen response early: NES = 1.827, *p* < 0.001, FDR < 0.001; estrogen response late: NES = 1.895, *p* < 0.001, FDR < 0.001, Figure S1G).

### 3.3 Establishment Prognostic Gene Model and Survival Outcomes in GC

**FIGURE 5:**
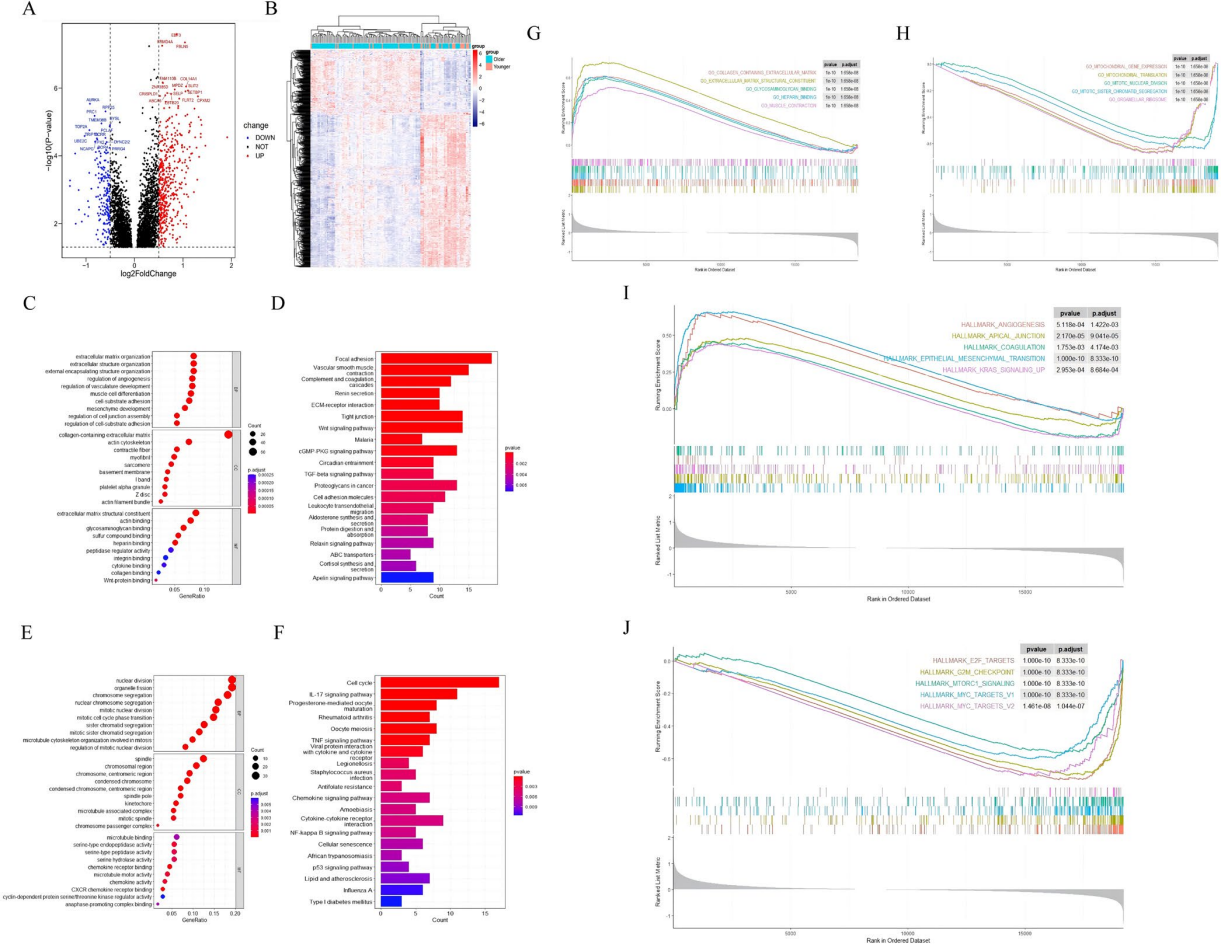
Functional annotation of DEGs in female GC patients of the discovery cohort. (A) Volcano plot of the DEGs with the top 15 DEGs. (B) Heat map of the DEGs. (C and D) GO function, KEGG pathway analysis of upregulated DEGs. (E and F) GO function, KEGG pathway analysis of downregulated DEGs. (G) Enrichment plots from GSEA in C5 collection of upregulated genes. (H) Enrichment plots from GSEA in C5 collection of downregulated genes. (I) Top 5 pathways associated with tumor progression in younger female GC patients. (J) Top 5 pathways associated with tumor progression in older female GC patients.

We further used GSE84437 cohort to construct a prognostic gene model for predicting the OS of female GC patients. The DEGs of the GSE84437 cohort were identified between 30 younger and 107 older female GC patients, including 463 upregulated genes and 184 downregulated genes (Figure 5A,B).

The enrichment analysis of GO and KEGG was performed on the DEGs of GSE84437. The results of GO analysis were shown in Figure 5C,E. GO analysis indicated that in BP, extracellular matrix organization, extracellular structure organization, external encapsulating structure organization, regulation of angiogenesis, and regulation of vasculature development were mainly enriched in younger female patients, while nuclear division, organelle fission, chromosome segregation, nuclear chromosome segregation, and mitotic nuclear division were mainly enriched in older female patients. As a result of KEGG, younger female patients showed significant enrichment in pathways related to cancer progression, such as focal adhesion, vascular smooth muscle contraction, complement and coagulation cascades, ECM–receptor interaction, and tight junction. However, older female patients showed main enrichment in cell cycle pathways, such as cell cycle, IL‐17 signaling pathway, progesterone‐mediated oocyte maturation, and oocyte meiosis. Enrichment results from GSEA in the C5 collection showed similar results to GO and KEGG analysis (Figure 5G,H). The top five pathways associated with tumor progression in younger female patients were EMT, apical junction, KRAS signaling, angiogenesis, and coagulation (Figure 5I). In older female patients, the top five pathways associated with tumor progression were E2F targets, G2/M checkpoint, mTORC1 signaling, MYC targets V1 and V2 (Figure 5J).

**FIGURE 6:**
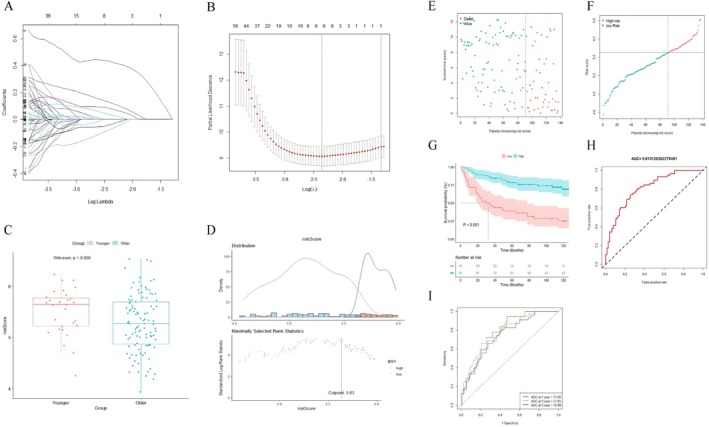
Relationship between risk score and prognosis in female GC patients. (A) Six gene expression signatures based on DEGs were selected by the LASSO Cox models. (B) Cross‐validation for tuning parameter selection in the LASSO model. (C) The distribution of risk scores in younger and older female patients. (D) The optimal cutoff based on maximally selected rank statistics. (E) The survival status of female patients (ranked by increasing risk score). (F) The distribution of risk scores in total female patients. (G) Kaplan–Meier curves of survival for different risk groups. (H) ROC curve of the risk score model. (I) Time‐dependent ROC curve of the risk score model for predicting 1, 3, and 5 years.

After further analysis of univariate Cox regression in the DEGs, we identified 266 genes related to survival time (*p* < 0.05). As a next step, survival‐related DEGs were subjected to Lasso Cox regression analysis, and six genes were finally identified and involved in the construction of the prognostic gene model, including NREP, GAD1, SLCO4A1, KRT17, DEFB1, and P3H2 (Figure 6A,B). Based on the results of the Lasso Cox regression analysis, the prognostic gene model was constructed as follows: Risk score = Exp(NREP) × (0.425262001) + Exp(GAD1) × (−0.033627453) + Exp(SLCO4A1) × (−0.066757311) + Exp(KRT17) × (0.119705227) + Exp(DEFB1) × (0.032480849) + Exp(P3H2) × (0.076266766).

After further analysis of applying risk score, there was a significant difference in the risk score between younger female GC patients and older female GC patients (*p* = 0.026, Figure 6C). The optimal cutoff was identified to classify female GC patients into two groups (high‐risk and low‐risk groups) with the most distinct survivals by a method based on maximally selected rank statistics (Figure 6D). The result revealed that the perfect ratio of high‐risk/low‐risk seemed to be 1:2, and female GC patients in the discovery cohort were well dispersed in the high‐risk group (*n* = 46) and low‐risk group (*n* = 91). The distribution plot of the risk score demonstrated that the survival times were reduced while the risk score increased (Figure 6E,F). Kaplan–Meier survival curves comparing high‐risk and low‐risk patients were also constructed to further evaluate the prognostic potential of the prognostic gene model. The result suggested that high‐risk patients showed worse OS compared with low‐risk patients (*p* < 0.01, Figure 6G). ROC analyses indicated that the prognostic value (AUC) of the prognostic gene model for predicting OS was 0.81 (Figure 6H). Moreover, the AUC values of this prognostic gene model for the 1‐year, 3‐year, and 5‐year OS of female patients with GC were 0.78, 0.78, and 0.75, respectively (Figure 6I). In conclusion, the prognostic gene model exhibited high prognostic value in the discovery cohort GSE84437.

### 3.4 Validation of the Prognostic Gene Model

**FIGURE 7:**
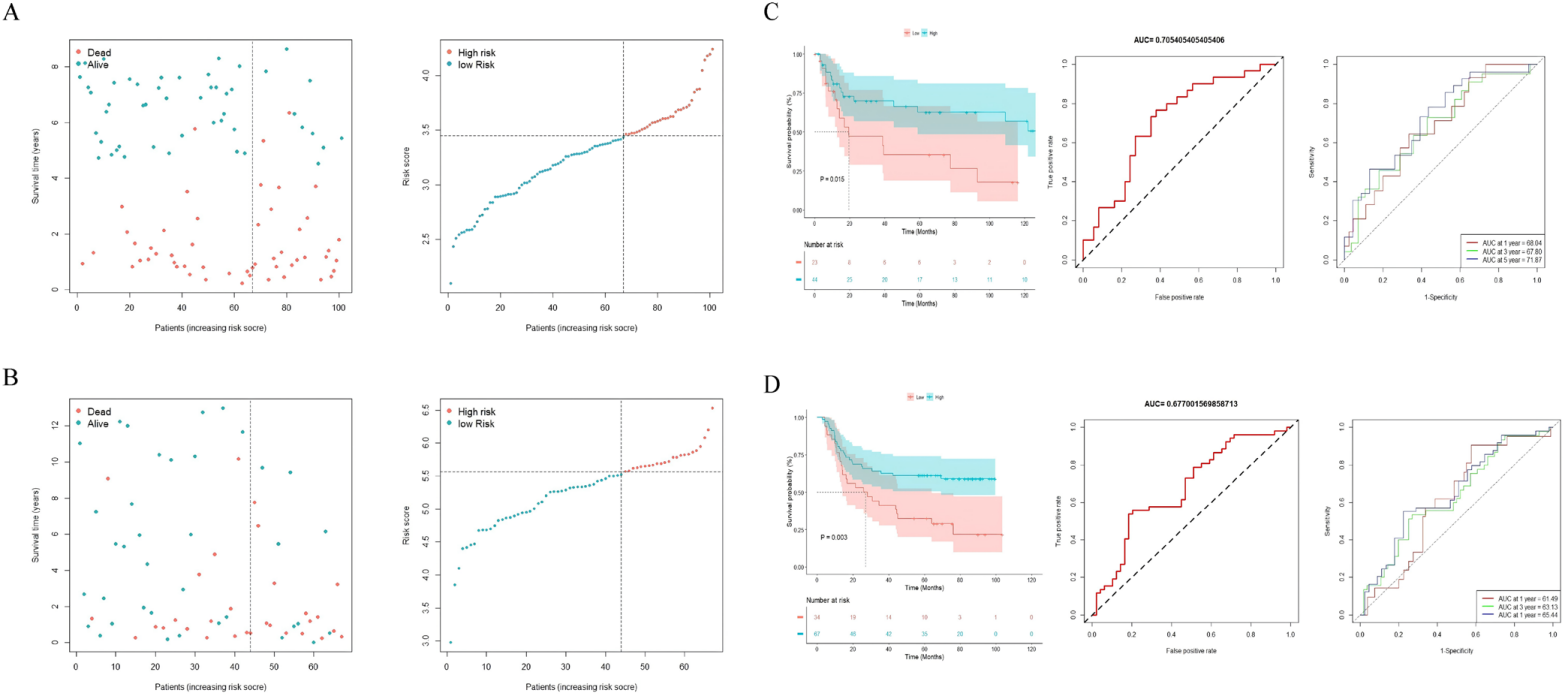
The results from external validation cohorts. (A) The distribution of survival status and risk scores in female patients of GSE15459. (B) The distribution of survival status and risk scores in female patients of GSE62254. (C) Kaplan–Meier curves and ROC curve from GSE15459. (D) Kaplan–Meier curves and ROC curve from GSE62254.

Two independent cohorts were used for external validations (GSE15459 and GSE62254). The patients of two validation cohorts were assigned into different risk subgroups based on the unified ratio consistent with the discovery cohort. The risk plot of the prognostic gene model indicated that as risk score increased, OS time decreased while mortality rose (Figure 7A,B). As illustrated in Figure 7C,D, low‐risk patients had a better OS than high‐risk patients whether in the GSE15459 cohort or the GSE62254 cohort (GSE15459, *p* = 0.015; GSE62254, *p* = 0.003, respectively). The AUC values of the prognostic gene model in the GSE15459 cohort and the GSE62254 cohort were 0.71 and 0.68, respectively. Furthermore, we estimated the AUC values for predicting OS at 1‐year, 3‐year, and 5‐year in the GSE15459 cohort and the GSE62254 cohort, respectively (GSE15459, 1‐year: 0.68, 3‐year: 0.68, 5‐year: 0.72; GSE62254, 1‐year: 0.61, 3‐year: 0.63, 5‐year: 0.65). As shown in Figure 7C,D, the AUC values were as expected, implying this prognostic gene model was an effective instrument for the prognostic risk classification of female GC patients.

To determine the independent prognostic value of prognostic gene model for female GC patients, we performed univariate Cox regression analysis and multivariate Cox regression analysis to explore prognostic independence of multiple clinical factors. Table 2 showed the result of univariate Cox regression analysis in discovery cohort, which suggested the risk score was significantly associated with the prognosis of GC in female patients (HR = 6.601, 95% CI: 3.630–12.000, *p* < 0.001). After further multivariate Cox regression analysis, Table 3 showed that the risk score presented as an independent prognostic factor after adjusting for other clinicopathologic characteristics (HR = 5.769, 95% CI: 3.011–11.053, *p* < 0.001). Based on the same analysis performed in external validations, the results were concordant with the findings available in the discovery cohort (GSE15459, HR = 2.171, 95% CI: 1.052–4.479, *p* = 0.036; GSE62254, HR = 3.136, 95% CI: 1.577–6.236, *p* = 0.001, Tables [Link], [Link]). Overall, the high‐risk patients’ prognosis was poorer compared to low‐risk patients.

**TABLE 2:** The result of univariate Cox regression analysis in discovery cohort.

Characteristics	Overall (*N* = 137)		*p*
	HR	95% CI	
Age	1.004	[0.9834, 1.026]	0.682
*T*			
1	0.4671	[0.06435, 3.3913]	0.4517
2	0.1986	[0.04823, 0.8174]	0.0251
3	0.5965	[0.26988, 1.3185]	0.2017
4	1		
*N*			
0	1		
1	4.082	[1.440, 11.57]	0.00813
2	6.03	[2.057, 17.68]	0.00106
3	5.152	[1.150, 23.08]	0.03214
Risk score	6.601	[3.63, 12]	< 0.001

**TABLE 3:** The result of multivariate Cox regression analysis in discovery cohort.

Characteristics	Overall (*N* = 137)		*p*
	HR	95% CI	
Age	1.0096	[0.9879, 1.032]	0.369458
*T*			
1	2.9545	[0.3581, 24.374]	0.3143
2	0.4796	[0.1105, 2.082]	0.3265
3	0.7856	[0.3469, 1.779]	0.5628
4	1		
*N*			
0	1		
1	3.4045	[1.1595, 9.996]	0.0258
2	3.8002	[1.2255, 11.784]	0.0208
3	5.0304	[1.0863, 23.295]	0.0388
Risk score	5.7688	[3.0108, 11.053]	< 0.001

The clinicopathologic characteristics of GC patients stratified by risk were listed in Table 4. Younger female GC patients were more likely to be in high‐risk group compared with the older (31.1% vs. 18.3%, *p* = 0.018). Nevertheless, when directly comparing the prognoses of younger and older female patients, we did not observe significant differences (*p* = 0.369, Figure S2). In addition, the stage of GC was more advanced in high‐risk group than that in low‐risk group (TNM I: 7.0% vs. 18.9%; TNM II: 17.5% vs. 29.7%; TNM III: 35.1% vs. 26.1%; TNM IV: 40.4% vs. 25.2%, *p* = 0.022).

**TABLE 4:** The clinicopathologic characteristics of female GC patients stratified by risk.

Characteristics	Overall (*N* = 305)		High risk (*N* = 103)		Low risk (*N* = 202)		*p*
	*N*, median	%, [IQR]	*N*, median	%, [IQR]	*N*, median	%, [IQR]	
Age (years)	63	[52.00, 70.00]	61	[48.80, 70.50]	63.15	[53.00, 70.00]	0.211
Group							
Younger	69	22.60%	32	31.10%	37	18.30%	0.018
Older	236	77.40%	71	68.90%	165	81.70%	
OS (days)	1740	[450.90, 2778.90]	750	[300.45, 2056.95]	2046.45	[689.48, 3240.00]	< 0.001
Lauren							
Intestinal	61	36.30%	17	29.80%	44	39.60%	0.453
Diffuse	102	60.70%	38	66.70%	64	57.70%	
Mixed	5	3.00%	2	3.50%	3	2.70%	
*T*							
1	4	1.70%	0	0.00%	4	2.50%	0.074
2	68	28.60%	16	20.00%	52	32.90%	
3	60	25.20%	23	28.70%	37	23.40%	
4	106	44.50%	41	51.20%	65	41.10%	
*N*							
0	41	17.20%	8	10.00%	33	20.90%	0.158
1	107	45.00%	37	46.20%	70	44.30%	
2	61	25.60%	25	31.20%	36	22.80%	
3	29	12.20%	10	12.50%	19	12.00%	
*M*							
0	87	86.10%	27	79.40%	60	89.60%	0.276
1	14	13.90%	7	20.60%	7	10.40%	
pStage							
1	25	14.90%	4	7.00%	21	18.90%	0.022
2	43	25.60%	10	17.50%	33	29.70%	
3	49	29.20%	20	35.10%	29	26.10%	
4	51	30.40%	23	40.40%	28	25.20%	

### 3.5 Establishment of a Nomogram to Predict Survival

**FIGURE 8:**
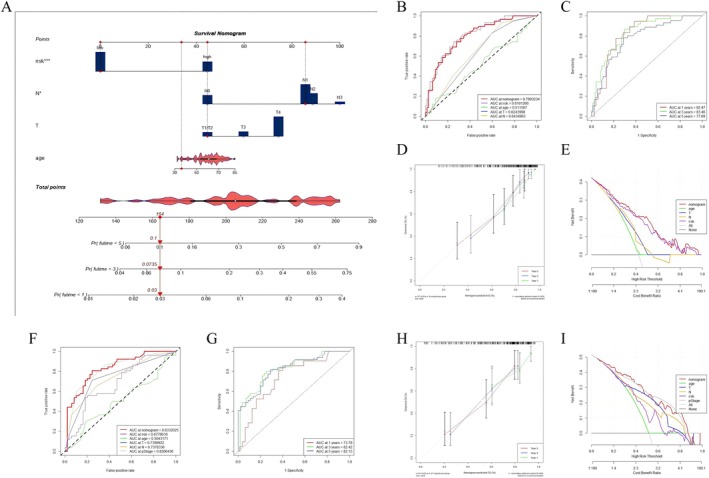
Construction and validation of a nomogram. (A) Nomogram for predicting 1, 3, and 5 years OS of female GC patients in the discovery cohort. (B) ROC curves of the nomograms in the discovery cohort. (C) Time‐dependent ROC curve of the nomogram in the discovery cohort for predicting 1, 3, and 5 years. (D) Calibration curves of the nomogram for predicting 1, 3, and 5 years OS in the discovery cohort. (F–I) The validation of the GSE62254 cohorts.

Due to the high correlation between prognostic gene model and patients’ prognosis, by integrating the risk scores and well‐known prognostic factors, a nomogram was constructed by using the discovery cohort for OS prediction. This nomogram was developed to predict 1‐year, 3‐year, and 5‐year OS rates in female patients with GC (Figure 8A). For the discovery cohort, the AUC values were as expected, implying this nomogram had an excellent predictive ability for prognosis (Figure 8B,C). The calibration curve showed well performance for the nomogram between actual observations and predicted values (Figure 8D). The clinical usefulness of the nomogram was quantified by the decision curve, and we found that this prognostic model with diverse clinical factors presented more net benefits for predicting the prognosis (Figure 8E). As shown in Figure 8F–I, similar results were shown in the GSE62254 cohort.

### 3.6 Assessment of the TME and Drug Sensitivity Analysis

**FIGURE 9:**
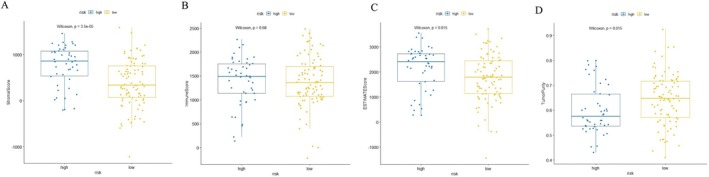
The assessment of TME in female GC patients from the discovery cohort. (A–D) Stromal score, immune score, ESTIMATE score, and tumor purity in GSE84437.

TME played an important role in tumor prevention and therapy. The risk score of the prognostic gene model was positively linked to the stromal score (*p* < 0.001, Figures 9A, S2E, and S3A). Meanwhile, this result was consistent with the GO analysis and GSEA results of younger female GC patients in the discovery cohort (Figure 5C,G), because the stromal cells, such as cancer‐associated fibroblasts, could secrete a variety of extracellular matrix components. However, the results revealed that the risk score was not associated with the immune score in either the discovery or two external validation cohorts (all *p* > 0.05, Figures 9B, S2F, and S3B).

**FIGURE 10:**
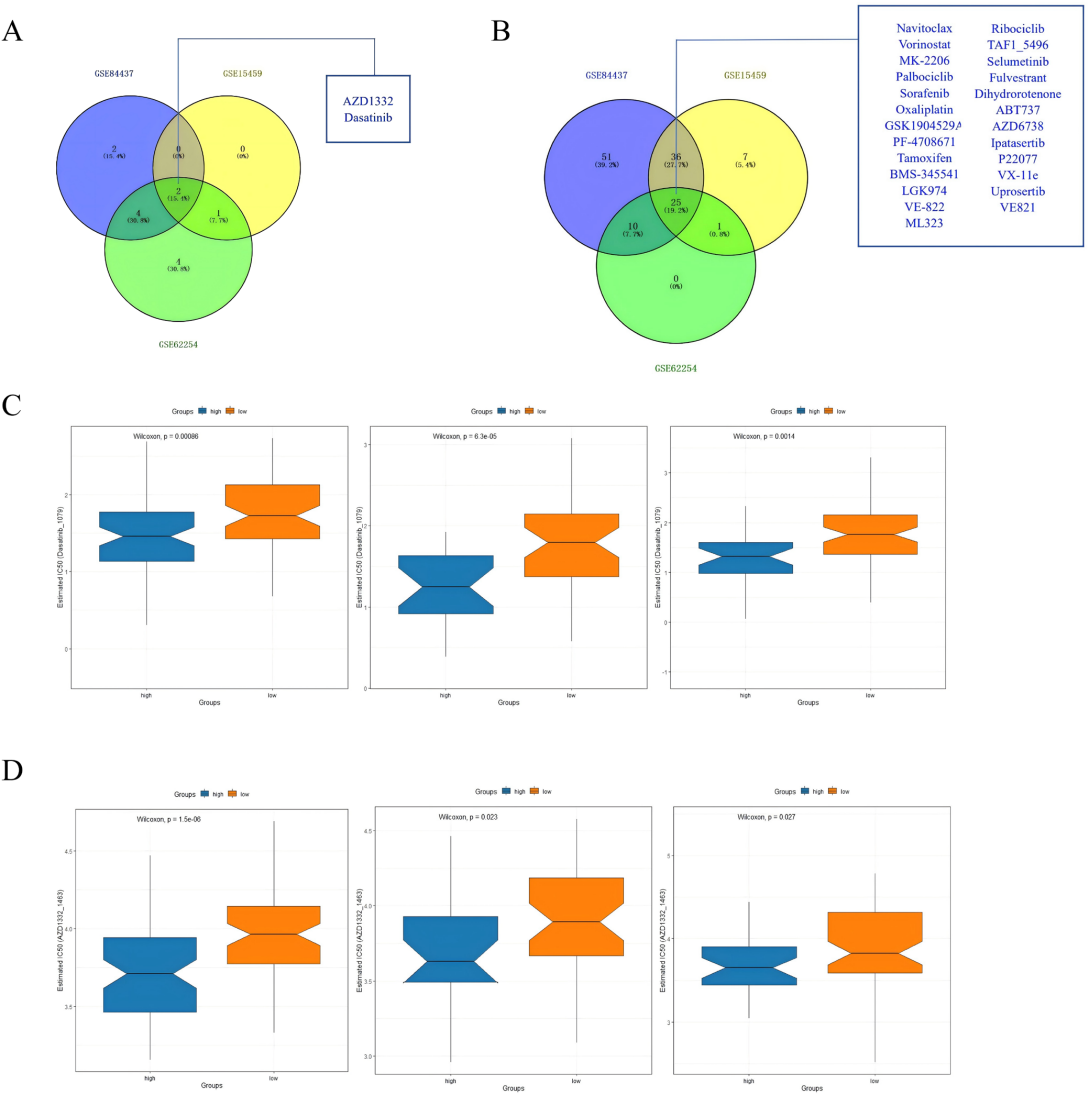
Drug sensitivity analysis of total female GC patients. (A) Sensitive drugs of the high‐risk group. (B) Resistant drugs of the high‐risk group. (C) Relationships between risk score and the sensitivity of AZD1332. (D) Relationships between risk score and the sensitivity of Dasatinib.

To explore suitable drugs for high‐risk group, we estimated the IC50 values of 198 drugs in GSE84437, GSE15459, and GSE62254 patients. All results of drug sensitivity analysis for each cohort were exhibited in Tables [Link], [Link]. We discovered that female GC patients with high‐risk might positively react to Dasatinib (targeting drug, ABL, SRC, Ephrins, PDGFR, and KIT inhibitor) and AZD1332 (targeting drug, receptor tyrosine kinase inhibitor; Dasatinib: GSE84437: *p* < 0.001, GSE15459: *p* < 0.001, and GSE62254: *p* = 0.001; AZD1332: GSE84437: *p* < 0.001, GSE15459: *p* = 0.023, and GSE62254: *p* = 0.027, Figure 10A,C,D). Female GC patients from high‐risk group exhibited greater resistance to 25 drugs, including those of Navitoclax (targeting drug, Bcl‐2 inhibitor), Vorinostat (targeting drug, HDAC inhibitor), MK‐2206 (targeting drug, AKT inhibitor), Palbociclib (targeting drug, CDK4/6 inhibitor), Sorafenib (targeting drug, PDGFR, KIT, VEGFR, and RAF inhibitor), Oxaliplatin (Chemotherapy drug), GSK1904529A (targeting drug, IGF1R, IR inhibitor), PF‐4708671 (targeting drug, S6K1 inhibitor), Tamoxifen (Chemotherapy drug), BMS‐345541 (targeting drug, IKK inhibitor), LGK974 (targeting drug, PORCN inhibitor), VE‐822 (targeting drug, ATR inhibitor), ML323 (targeting drug, USP1, UAF1 inhibitor), Ribociclib (targeting drug, CDK4/6 inhibitor), TAF1_5496 (targeting drug, TAF1 inhibitor), Selumetinib (targeting drug, MEK1/2 inhibitor), Fulvestrant (targeting drug, ESR inhibitor), Dihydrorotenone (mitochondrial inhibitor), ABT737 (targeting drug, Bcl‐2 inhibitor), AZD6738 (targeting drug, ATR inhibitor), Ipatasertib (targeting drug, AKT inhibitor), P22077 (targeting drug, USP7/47 inhibitor), VX‐11e (targeting drug, ERK2 inhibitor), Uprosertib (targeting drug, AKT inhibitor), and VE821 (targeting drug, ATR inhibitor), than those from low‐risk group patients (Figure 10B). Overall, these results indicated that the prognostic gene model was correlated with drug sensitivity.

## 4 Discussion

In this study, we investigated the molecular characteristics of female GC patients on the basis of six GEO cohorts, suggesting that the pathogenesis mechanism of GC in female patients was associated with estrogen. Furthermore, compared with older female patients, we found aldosterone and relaxin pathways were concentrated in the younger group. The most critical finding in the present study was that the high‐risk group showed worse OS compared with the low‐risk group using the prognostic gene model we created. Compared with older female patients, younger female patients had a higher tendency to be in the high‐risk group. To the best of our knowledge, our study was the first to investigate the comprehensive molecular profiles of younger female GC patients, which manifested the mechanisms of pathogenesis and prognosis in younger females with GC.

Previous prospective cohort studies and human cell line studies suggested that female hormones might play a protective role in GC risk [25, 26, 27, 28, 29, 30, 31]. However, the occurrence of GC was frequently observed that females were more susceptible than males in the younger group, which prompted a reconsideration of the potential mechanisms of estrogen [4, 5, 6, 7, 8, 9, 10, 11]. In our study, with the exception of other cancer‐ and metastasis‐associated pathways, female GC patients were markedly enriched in estrogen response early and late with GSEA enrichment analysis, which suggested there was a potential relationship between GC and estrogen pathways. Notably, when we used GSEA enrichment analysis to evaluate potential mechanism differences between younger and older female GC patients, estrogen response early and late were enriched in both groups. This finding suggested that estrogen might not be the key influence in the susceptibility of younger female patients to develop GC.

As the results of KEGG analysis, we found there was a significant difference in enriched other hormone‐related pathways between younger group and older group. Aldosterone and relaxin pathways were concentrated in younger group, while growth hormone synthesis, secretion and action and thyroid hormone signaling pathway were concentrated in older group. There were many studies suggesting the relationship between these hormones and GC or other cancer [32, 33, 34, 35, 36, 37, 38, 39, 40, 41]. Aldosterone, a mineralocorticoid hormone, regulated electrolyte balance and blood pressure. It was hypothesized to act in gastric mucosal protection and repair, with chronic inflammation and damage being key steps in gastric carcinogenesis [32]. Relaxin, a peptide hormone active during pregnancy and childbirth, promoted smooth muscle relaxation and cervical softening. One study showed it could enhance ovarian cancer cell invasion and metastasis by degrading the extracellular matrix and altering cell adhesion [33]. Notably, another research indicated that relaxin's abnormal activation might enable GC to resist oxidative stress and drive their invasion, metastasis, and chemoresistance [35]. Growth hormone, secreted by the anterior pituitary, promoted growth and regulated metabolism. It was also found to regulate TME, EMT, DNA repair, tumor angiogenesis, and chemoresistance, thereby facilitating tumor initiation and progression [36]. In this study, it was observed that high growth hormone levels correlated with lower disease control rates in GC immunotherapy [36]. Another study indicated that growth hormone modulated GC growth and apoptosis via the PI3K/AKT signaling pathway [37]. Thyroid hormones played a crucial role in metabolism and development. Research demonstrated that thyroid hormone accumulation could lead to HIF‐α overexpression, which in turn promoted VEGF expression and enhanced glycolysis. Meanwhile, thyroid hormones were found to disrupt the tricarboxylic acid cycle and drive metabolic reprogramming in GC through the PIK/Akt signaling pathway [40]. Additionally, one study suggested that hypothyroidism had been associated with a trend toward prolonged OS [41]. However, pathway enrichment analysis had inherent limitations, and its results should be combined with other experimental verifications to more accurately determine their biological relevance and significance.

Most of the relevant clinical studies showed that younger female GC patients had a poorer prognosis compared to the older, however, the evidence regarding the mechanisms underlying such outcomes was scarce at the genetic level [4, 20, 21, 22, 23, 24]. In this study, we constructed the effective prognostic gene model containing 6 genes, namely NREP, GAD1, SLCO4A1, KRT17, DEFB1, and P3H2, and demonstrated its predictive ability. Although no significant difference was found in the prognosis when directly comparing younger and older female GC patients, we found that the younger female GC patients had higher risk scores compared with older female GC patients in the prognostic gene model we created. And patients in the high‐risk group had a much worse prognosis than those in the low‐risk group.

Most of the genes in the prognostic gene model have been reported to be associated with cancer development. NREP played an important role in the progression of GC through diverse mechanisms, such as EMT activation, cancer‐associated fibroblasts activation, actin cytoskeleton remodeling, and M2 macrophage infiltration, and its expression was powerfully associated with T stage and histologic grade [42, 43, 44]. KRT17 could promote the proliferation, migration, and invasion of GC cells, and its expression was positively correlated with the TMN stage, lymphatic metastasis, depth of invasion, and vascular invasion [45, 46, 47]. Moreover, they found KRT17 could regulate the cell cycle and modulate cell cycle proteins, suggesting that KRT17 might be a possible molecular target for targeted therapy in GC [46, 47]. Pignata et al. reported that P3H2 was a new molecular player involved in new vessel formation, and they found that P3H2 knockdown prevented pathological angiogenesis in vivo [48]. Pathological angiogenesis in GC might be associated with its overexpression. The expression of human β‐defensin 1 (encoded by DEFB1) was found to be associated with Hp infection, while the mechanisms by which DEFB1 achieved its effects in GC remained unclear [49, 50]. In our study, SLCO4A1 was a protective factor for the prognosis of female GC patients. However, most studies indicated the elevated expression of SLCO4A1 might facilitate the progression of cancer, as it mediated the cellular uptake of many substrates including hormones, which regulated the progression of cancer through binding the hormone receptors [51, 52, 53]. Yet, another study based on TCGA data also identified SLCO4A1 as a protective factor for GC prognosis. Thus, the mechanisms of SLCO4A1 in GC development required further exploration. GAD1 contributed to the progression in various types of cancer [54, 55, 56, 57], however, it was identified as a favorable gene for prognosis of female GC patients in our study. Currently, no relevant study has been conducted to investigate the role of GAD1 in GC.

Two critical genes in the prognostic gene model, NREP and KRT17, were all related to EMT, which suggested that EMT could be the main reason for the high‐risk group with poor prognosis. EMT is a reversible cellular process that transiently induces epithelial cells to quasi‐mesenchymal cell characteristics [58]. During carcinoma progression, EMT can increase metastatic powers and elevate therapeutic resistance of cancer [58]. Consistent with this, our study showed that the high‐risk group had a more advanced stage of GC and was resistant to more drugs than the low‐risk group.

The risk score calculated by the prognostic gene model correlated significantly with clinicopathologic characteristics of female GC patients. After controlling confounding parameters, the results indicated that the risk score was an independent predictor for female GC patients’ survival outcomes. To further improve the accuracy of prognostic prediction, we constructed and validated a nomogram by screening various indexes and made it easier to use the prognostic gene model.

TME, the environment in which the tumor resided, consisted of malignant cells, immune cells, stromal cells, extracellular matrix, and a variety of cytokines, and was nourished by a vascular network [59, 60]. Stromal cells were thought to have important roles in tumor growth, disease progression, and drug resistance, and the percentage of stromal cells in TME represents the stromal score [61, 62, 63, 64, 65]. Based on previous studies, GC patients with stromal score‐high showed poor OS and identified as an independent prognostic factor [64, 65]. Consistent with this finding, our study found that the risk score of prognostic gene model was positively linked to stromal score, which suggested the poor prognosis of the high‐risk group might be closely correlated with stromal cells and extracellular matrix in TME.

Whereas surgery remains a mainstay, multimodality treatment is the standard of care for advanced resectable GC, with particular emphasis on perioperative chemotherapy [66, 67, 68]. However, increasing evidence has shown that stromal cells, such as cancer‐associated fibroblasts, mediate chemotherapy resistance in several tumors by releasing cytokines, exosomes, and metabolites [69, 70, 71]. Thus, we explored the sensitivity of various drugs in female GC patients between two risk subgroups. In our study, we found that female patients of the high‐risk group were resistant to 25 drugs and sensitive to two drugs. Notably, we found that the high‐risk group was resistant to the ESR inhibitor Fulvestrant, which might provide new insights into the role of estrogen in female GC.

A key advantage of this study was that the significant difference in enriched hormone‐related pathways between younger and older groups was identified, and a prognostic gene model based on the DEGs between younger and older female patients was constructed. Another advantage of the current study was the consistent statistical results in three independent cohorts, confirming the robustness and precision of the prognostic gene model. The prognostic gene model could be integrated into clinical decision support systems to aid in risk stratification of female GC patients, enabling clinicians to promptly identify high‐risk female patients and develop tailored treatment plans. The identified gene set could serve as a biomarker panel for personalized medicine approaches. By targeting specific genes or pathways highlighted in our study, more effective and less toxic therapies could be developed. The limitations of this study included the fact that all gene expression and clinical data from public databases were obtained retrospectively, and inherent selection bias might affect the accuracy of the analysis results. Extensive prospective studies and complementary in vivo and in vitro experimental studies were necessary to gain insight into the potential mechanisms involved in GC development of younger female patients, thus confirming our findings. Further, given that the gene expression profiles for this study were derived from bulk tissue data, contamination from noncancer cells is unavoidable. Therefore, the proportion of stromal cells probably fluctuates across samples. Consequently, the gene expression of stromal cells might sway the enrichment of “EMT‐related genes” and other pathways linked to stromal cells in the high‐risk group.

## 5 Conclusion

In conclusion, we provided the comprehensive molecular profiles of younger female GC patients and found that there was a significant difference in enriched hormone‐related pathways between the younger group and the older group. Compared with older female patients, younger female patients were more likely to be in the high‐risk group, which showed worse OS than low‐risk patients.

## Author Contributions

**Xiaoyi Luan:** writing – original draft and data curation. **Lulu Zhao:** writing – original draft and visualization. **Wanqing Wang:** formal analysis. **Penghui Niu:** formal analysis. **Xue Han:** data curation. **Zerong Wang:** resources. **Xiaojie Zhang:** writing – review and editing. **Dongbing Zhao:** writing – review and editing and supervision. **Yingtai Chen:** conceptualization, resources, methodology, writing – review and editing, supervision. All authors discussed the findings and approved the final version of the manuscript.

## Funding

This work was supported by the grant from National Key R&D Program of China (2017YFC0908300) and 2023 Scientific Research Project of Chronic Diseases Control and Health Education (BJMB0012023024005).

## Ethics Statement

All data can be found in GEO databases. Ethical approval has been obtained for this study.

## Consent

The authors have nothing to report.

## Conflicts of Interest

The authors declare no conflicts of interest.

## Supporting information


**Figure S1:** Functional annotation of DEGs in female GC patients of the older group. (A and B) GO function, KEGG pathway analysis of upregulated DEGs. (C and D) GO function, KEGG pathway analysis of downregulated DEGs. (E) Enrichment plots from GSEA in C5 collection of upregulated genes. (F) Enrichment plots from GSEA in C5 collection of downregulated genes. (G) Endocrine‐related pathways in the enrichment plot from GSEA in HALLMARK collection.

**Figure S2:** The Kaplan–Meier curves for OS between younger and older female patients.

**Figure S3:** The assessment of TME in female GC patients from external validation cohorts. (A–D) Stromal score, immune score, ESTIMATE score, and tumor purity in GSE15459. (E–H) Stromal score, immune score, ESTIMATE score, and tumor purity in GSE62254.

**Table S1:** Univariate Cox regression of female GC patients in GSE15459.

**Table S2:** Multivariate Cox regression of female GC patients in GSE15459.

**Table S3:** Univariate Cox regression of female GC patients in GSE62254.

**Table S4:** Multivariate Cox regression of female GC patients in GSE62254.

**Table S5:** Drug sensitivity analysis of female GC patients in GSE84437.

**Table S6:** Drug sensitivity analysis of female GC patients in GSE15459.

**Table S7:** Drug sensitivity analysis of female GC patients in GSE62254.

## Data Availability

The public datasets analyzed in this study can be found in GSE (https://www.ncbi.nlm.nih.gov/geo/).
