# Supplementary figures and images for: Molecular Characteristics, Potential Mechanisms, and Prognostic Gene Model of Younger Female Patients With Gastric Cancer
Source: Cancer Rep (Hoboken). 2026 Mar 3;9(3):e70469. doi: 10.1002/cnr2.70469 (PMC12954549; doi:10.1002/cnr2.70469)

A

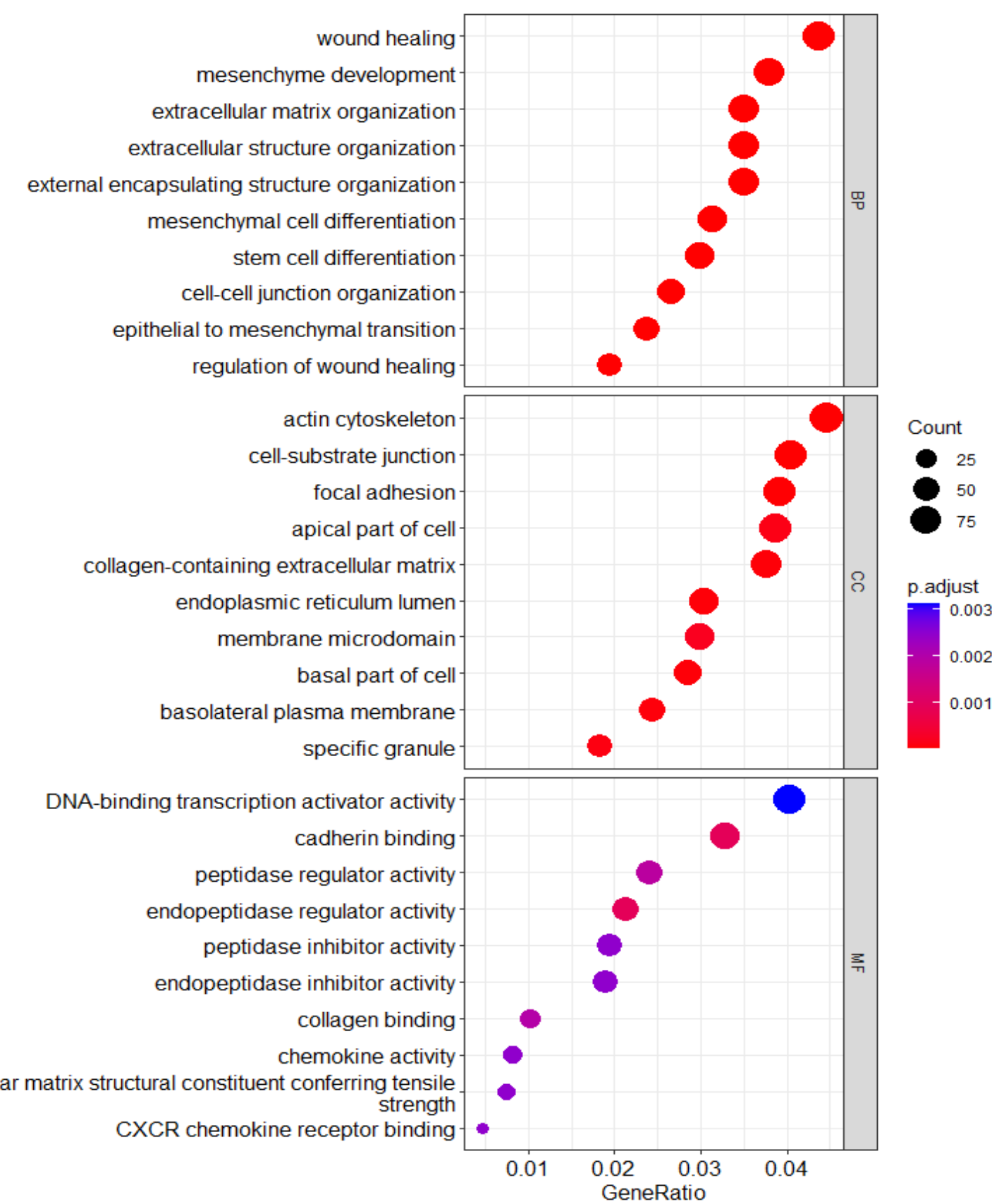

C

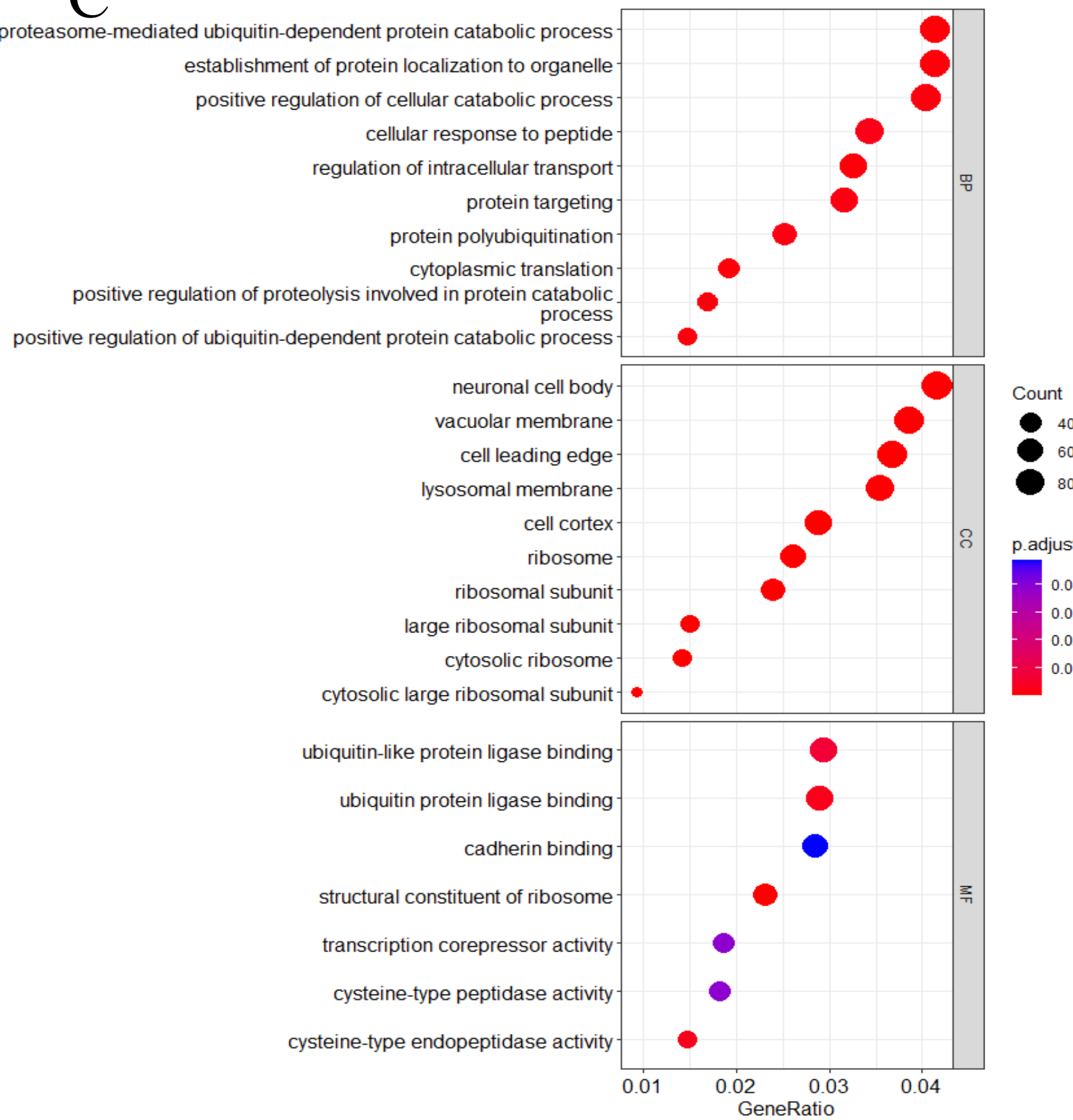

B

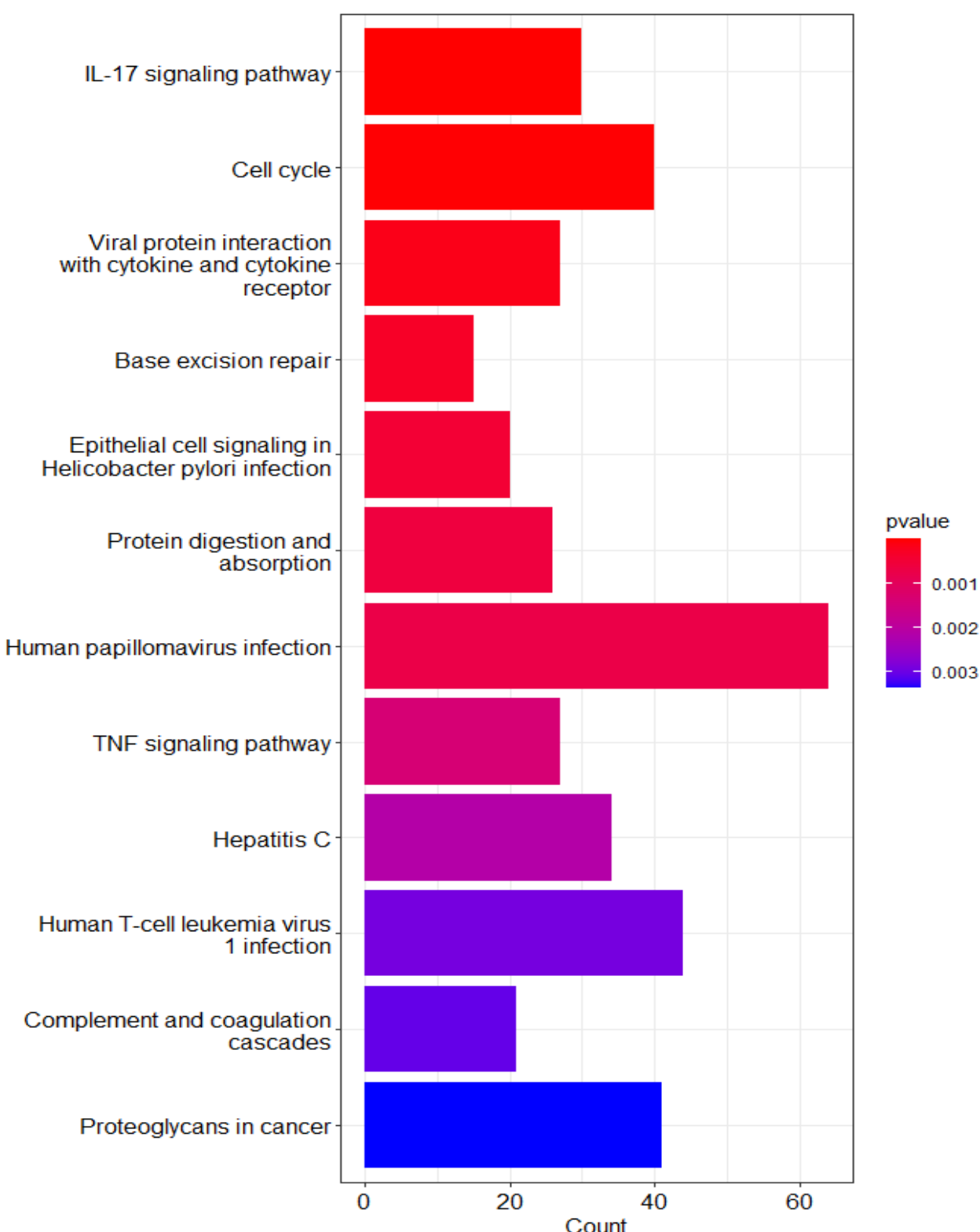

D

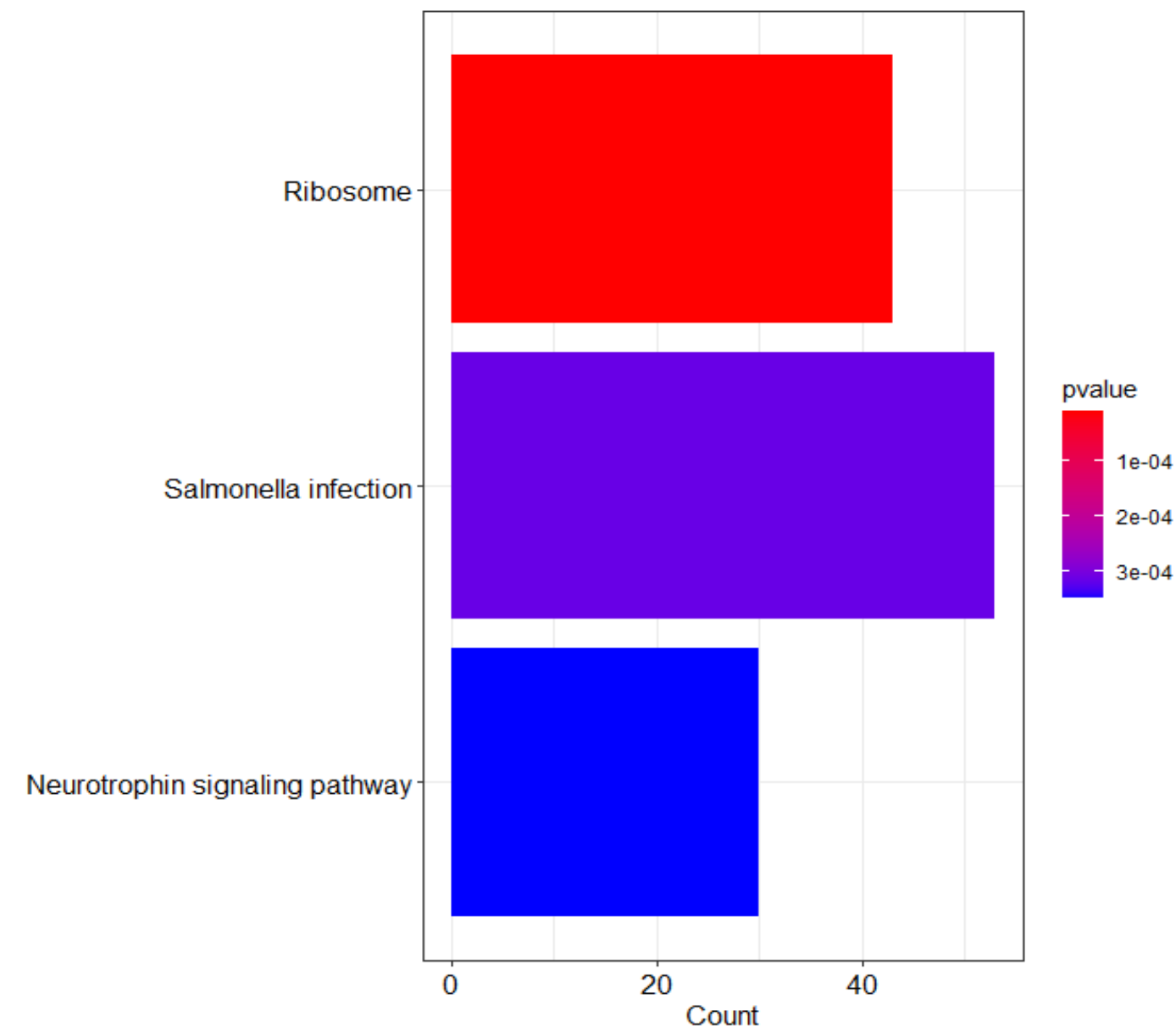

E

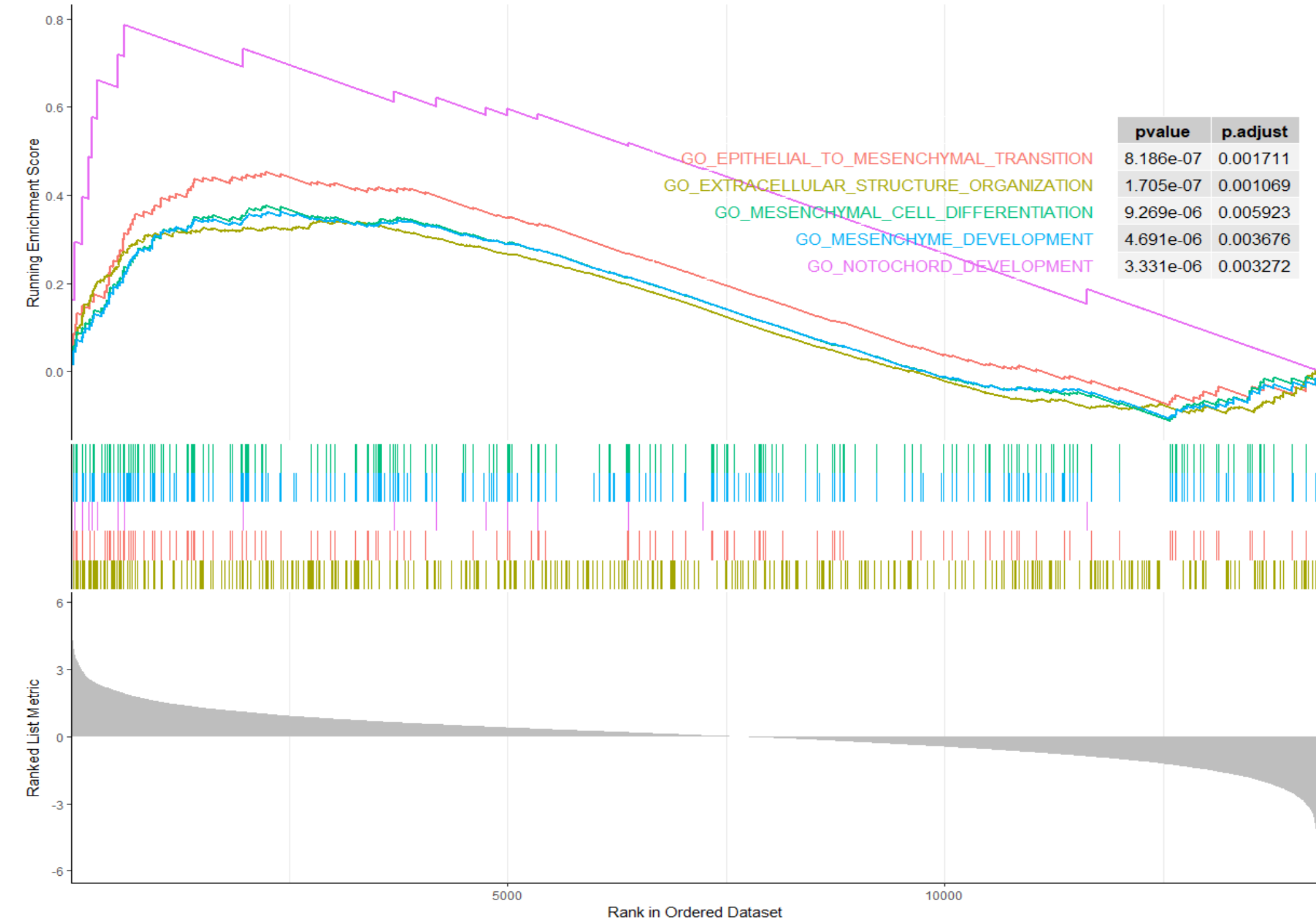

F

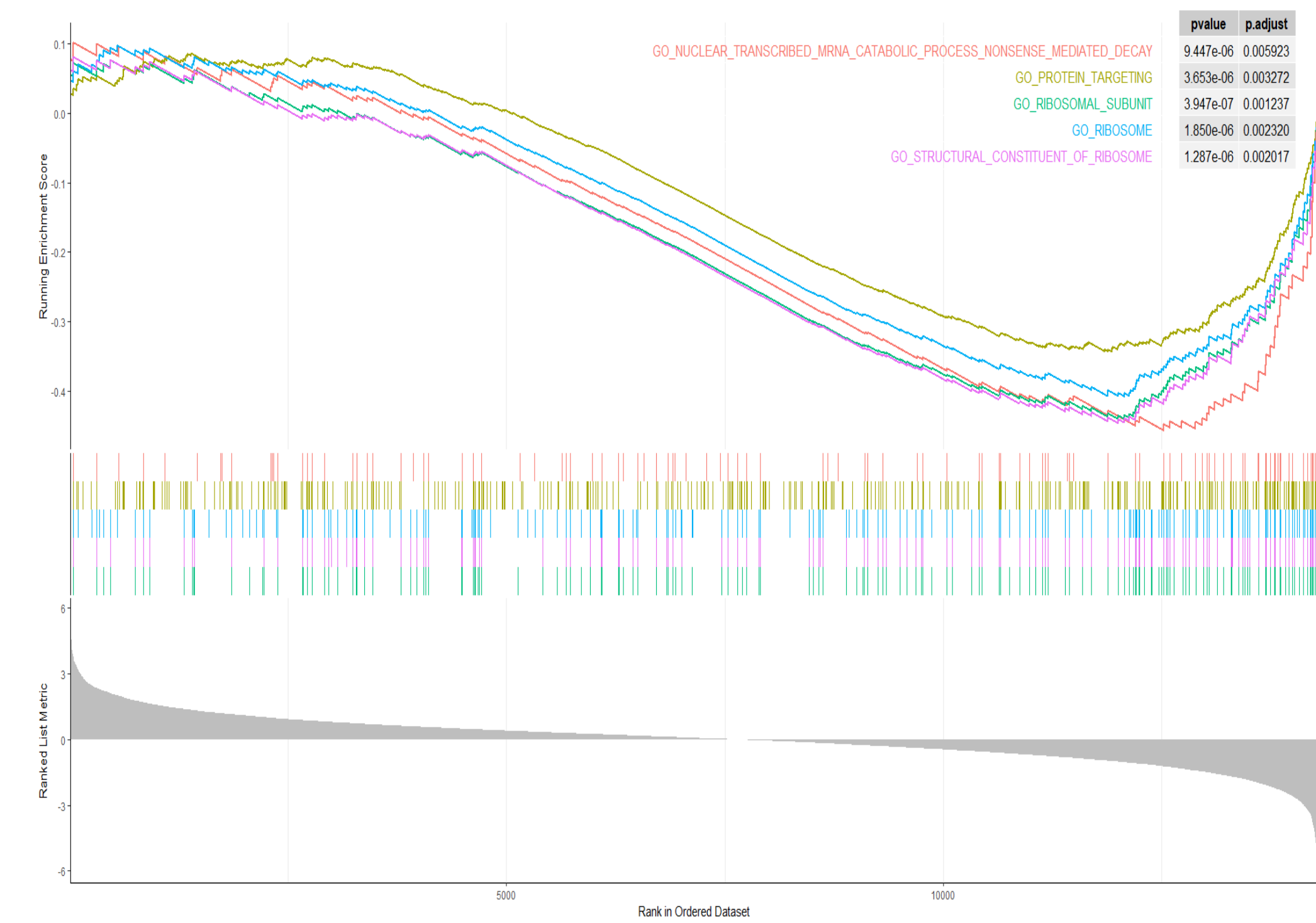

G

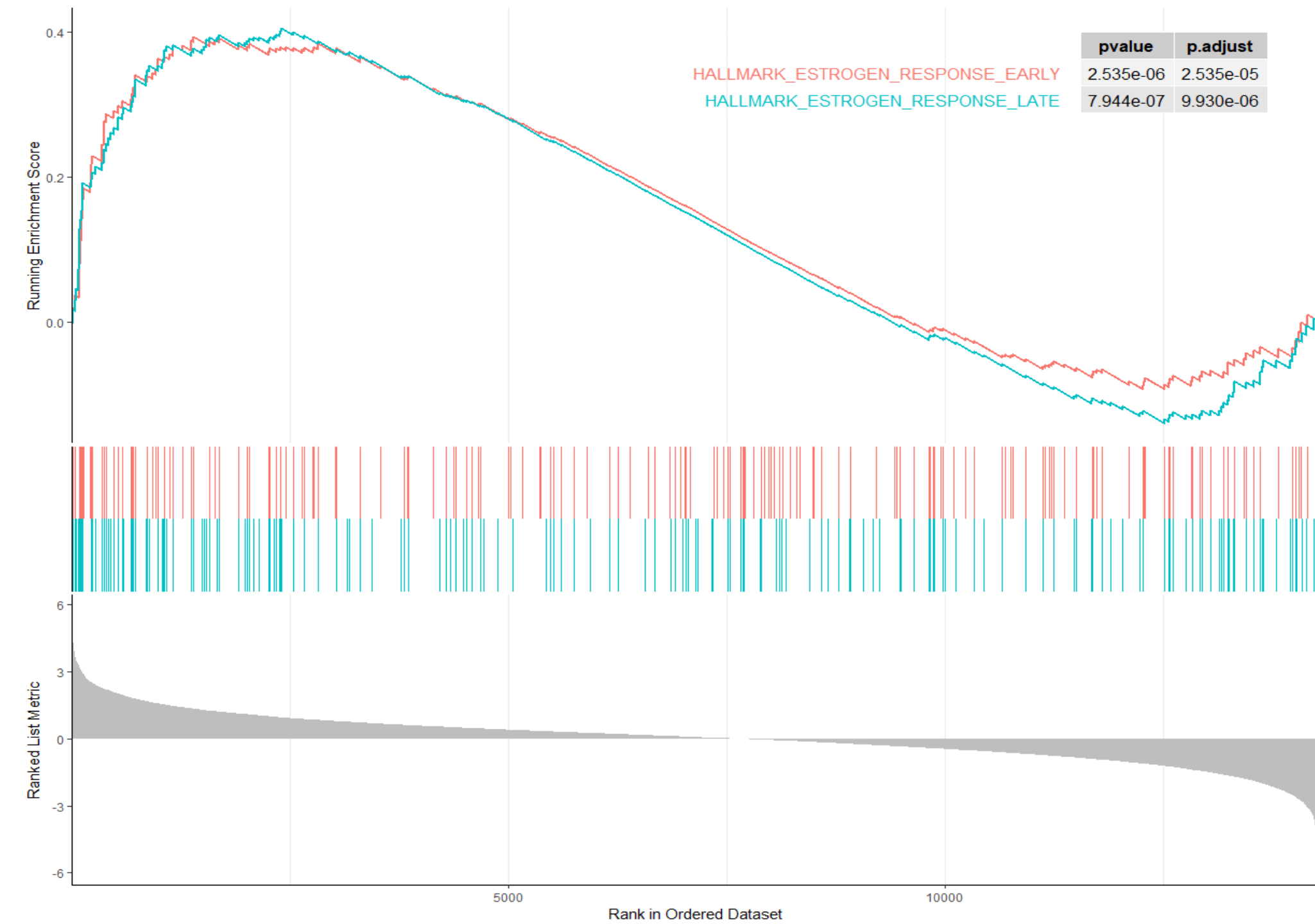

Supplement: Supplementary file 1 — Figure S1: Functional annotation of DEGs in female GC patients of the older group. (A and B) GO function, KEGG pathway analysis of upregulated DEGs. (C and D) GO function, KEGG pathway analysis of downregulated DEGs. (E) Enrichment plots from GSEA in C5 collection of upregulated genes. (F) Enrichment plots from GSEA in C5 collection of downregulated genes. (G) Endocrine‐related pathways in the enrichment plot from GSEA in HALLMARK collection. [file CNR2-9-e70469-s006.pdf]

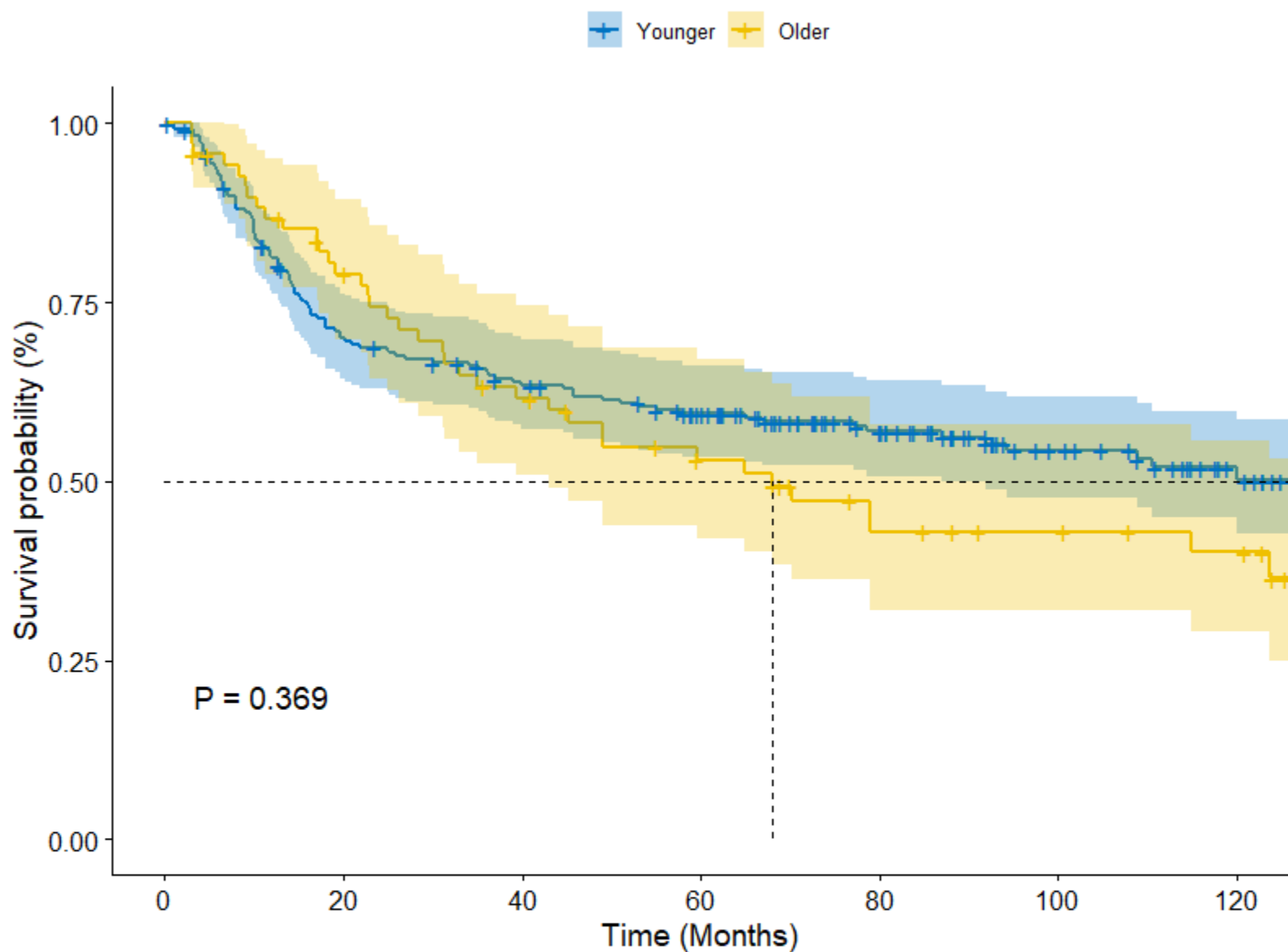

Number at risk

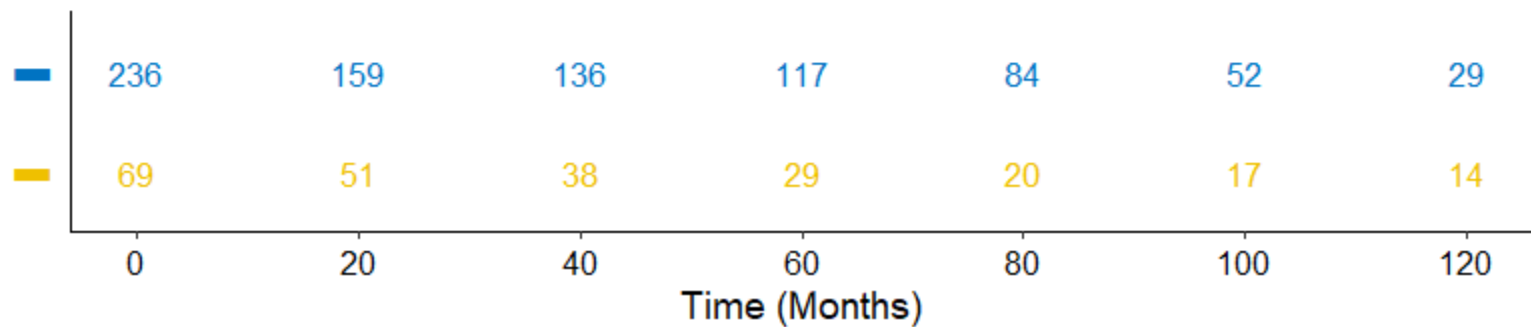

Supplement: Supplementary file 2 — Figure S2: The Kaplan–Meier curves for OS between younger and older female patients. [file CNR2-9-e70469-s005.pdf]

A

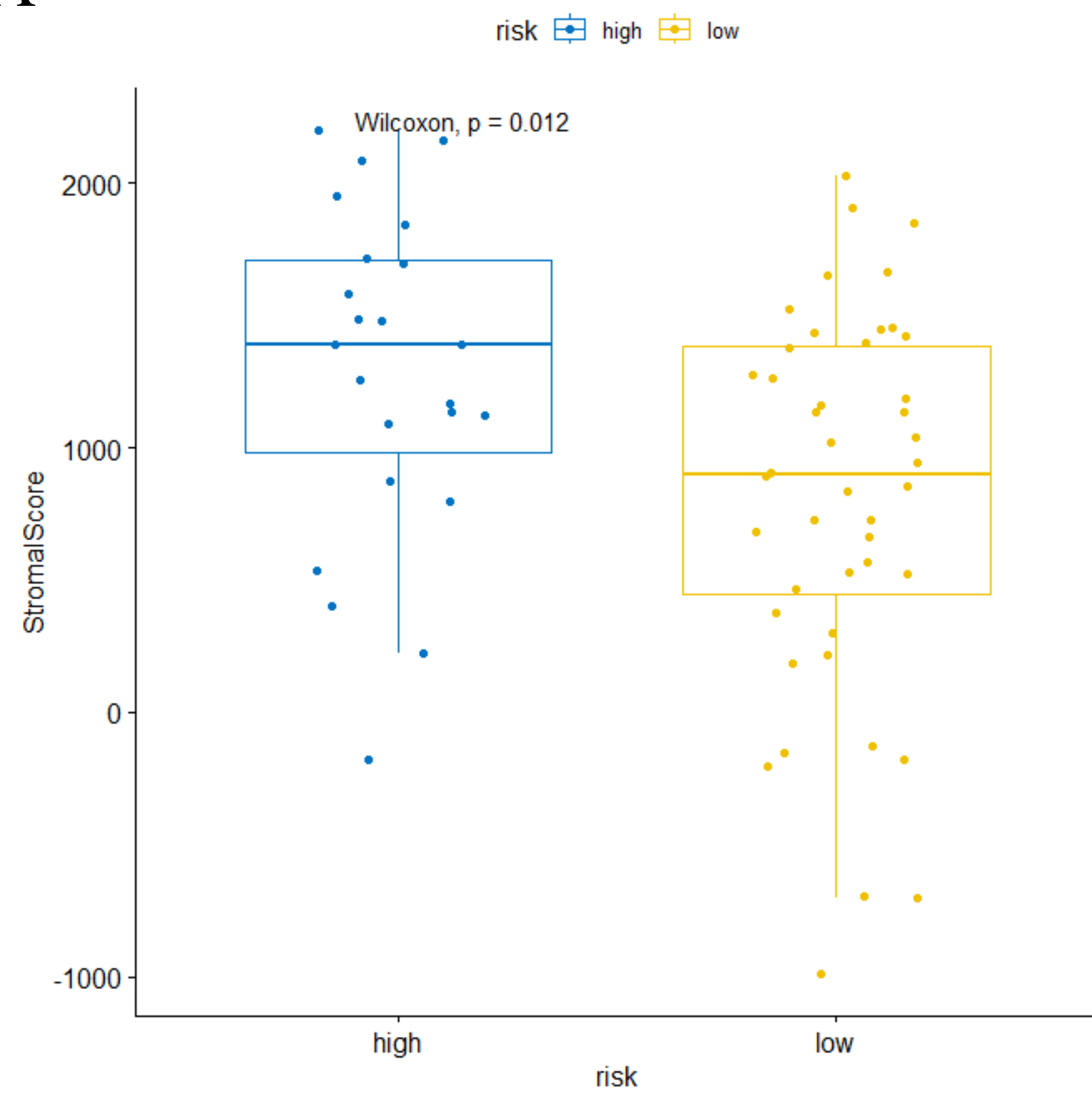

B

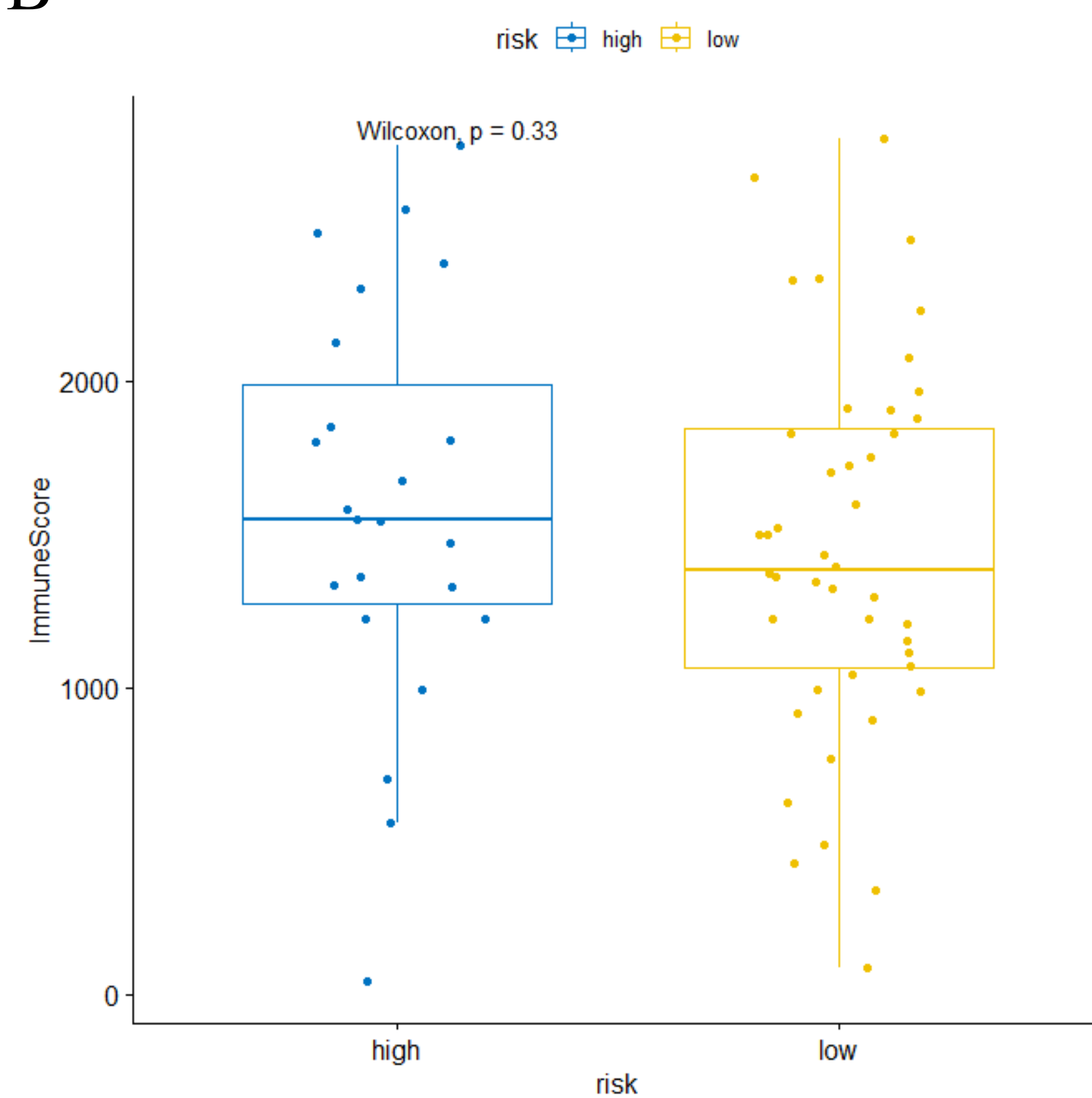

C

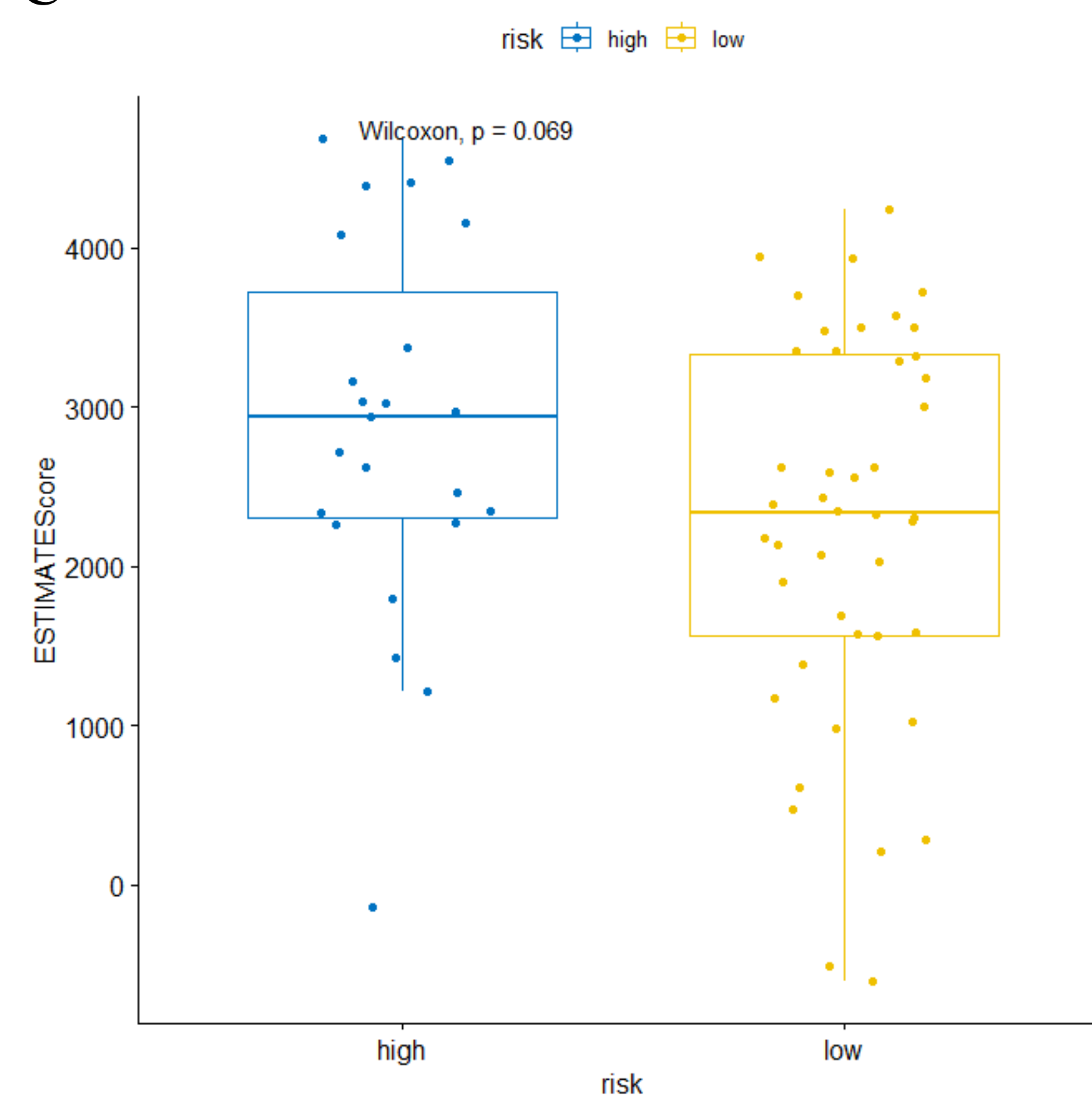

D

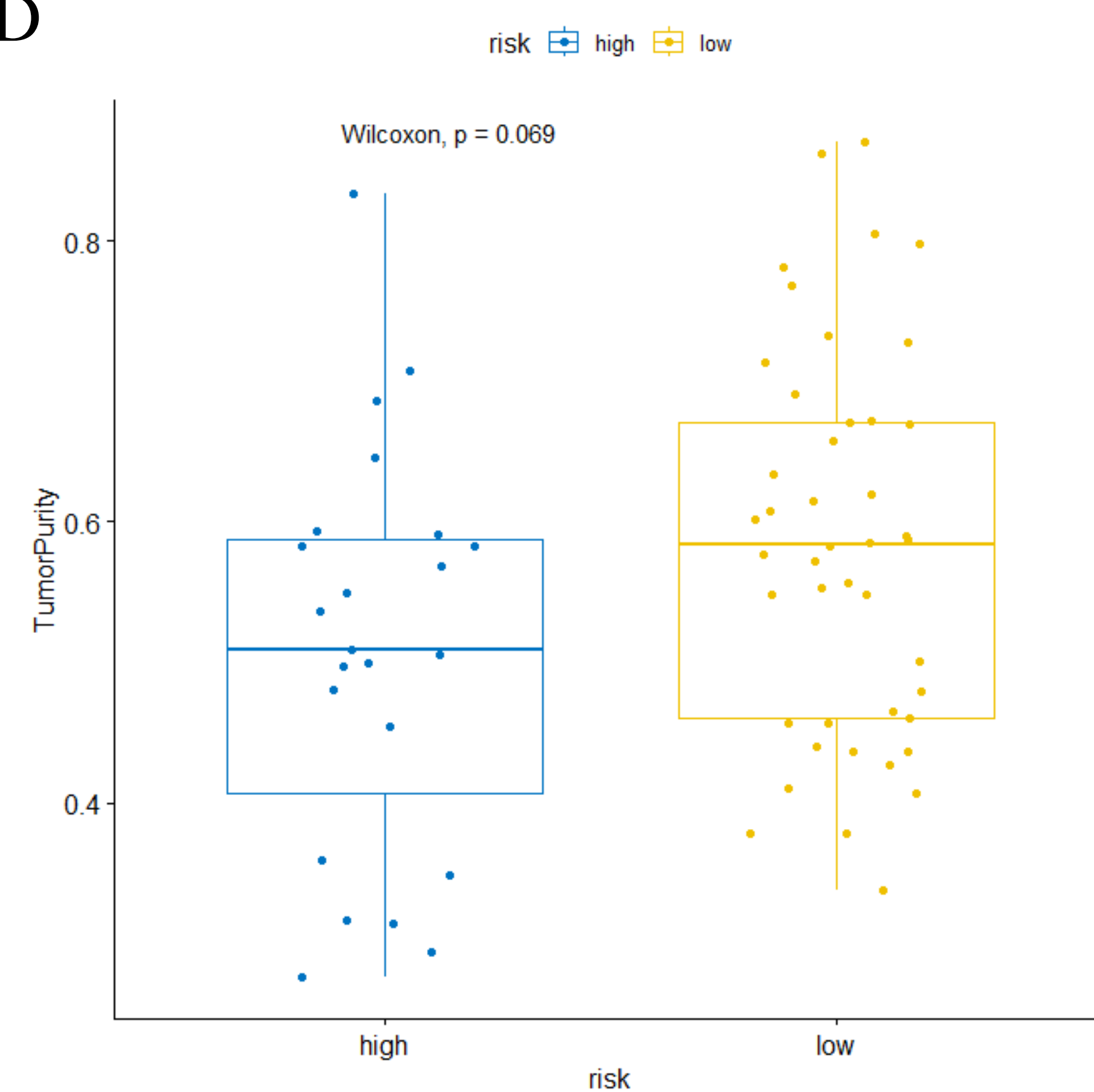

E

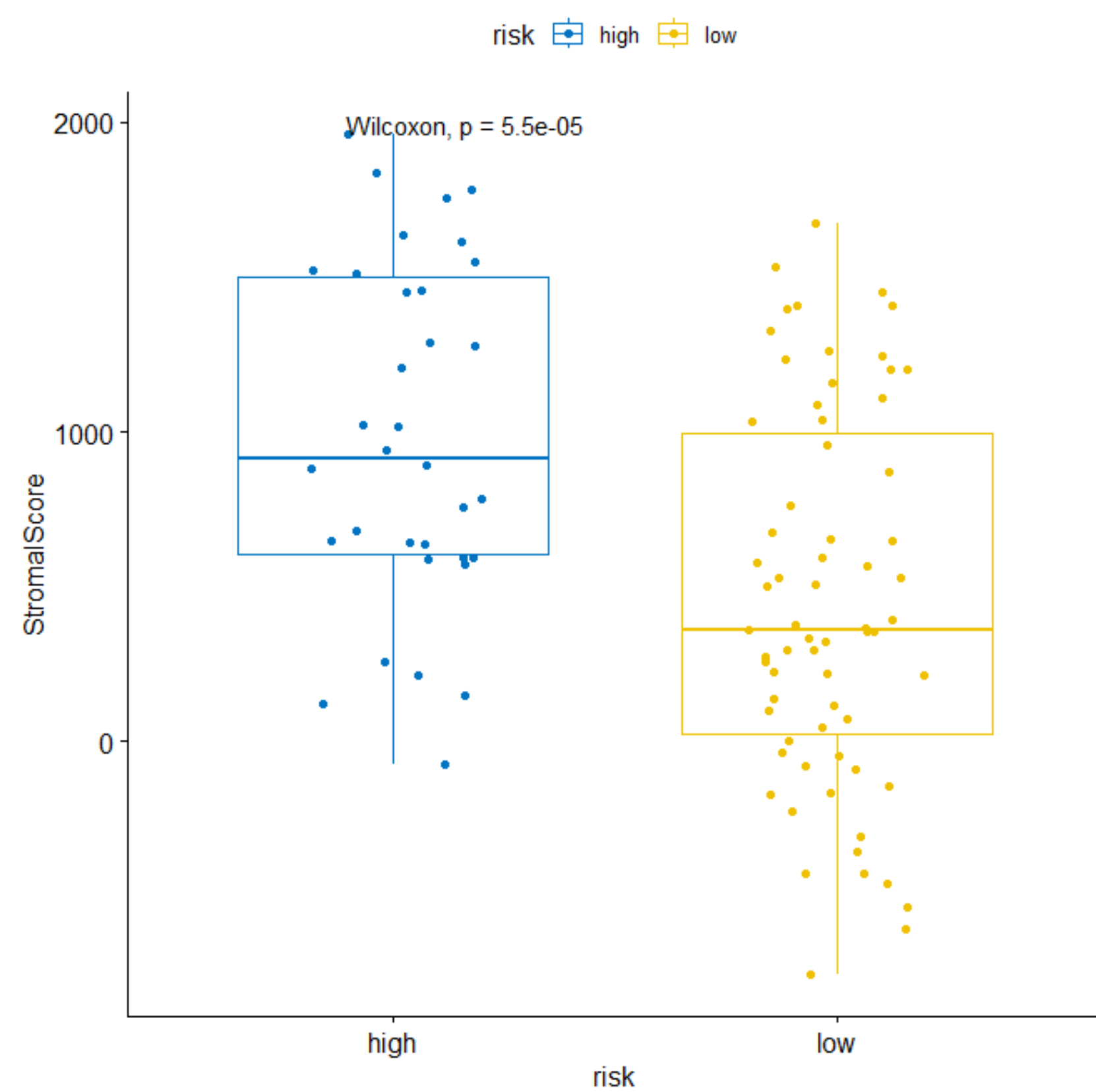

F

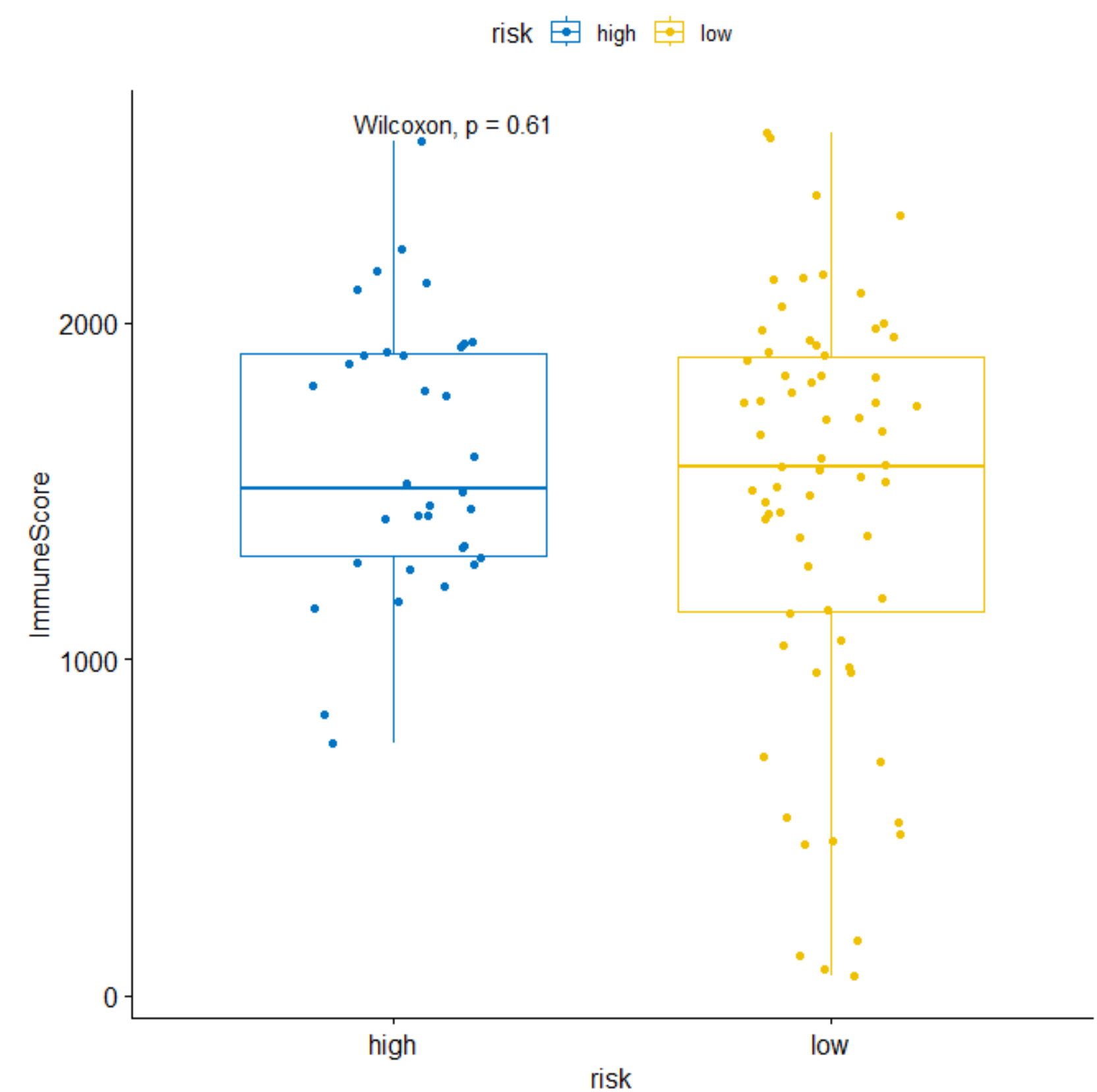

G

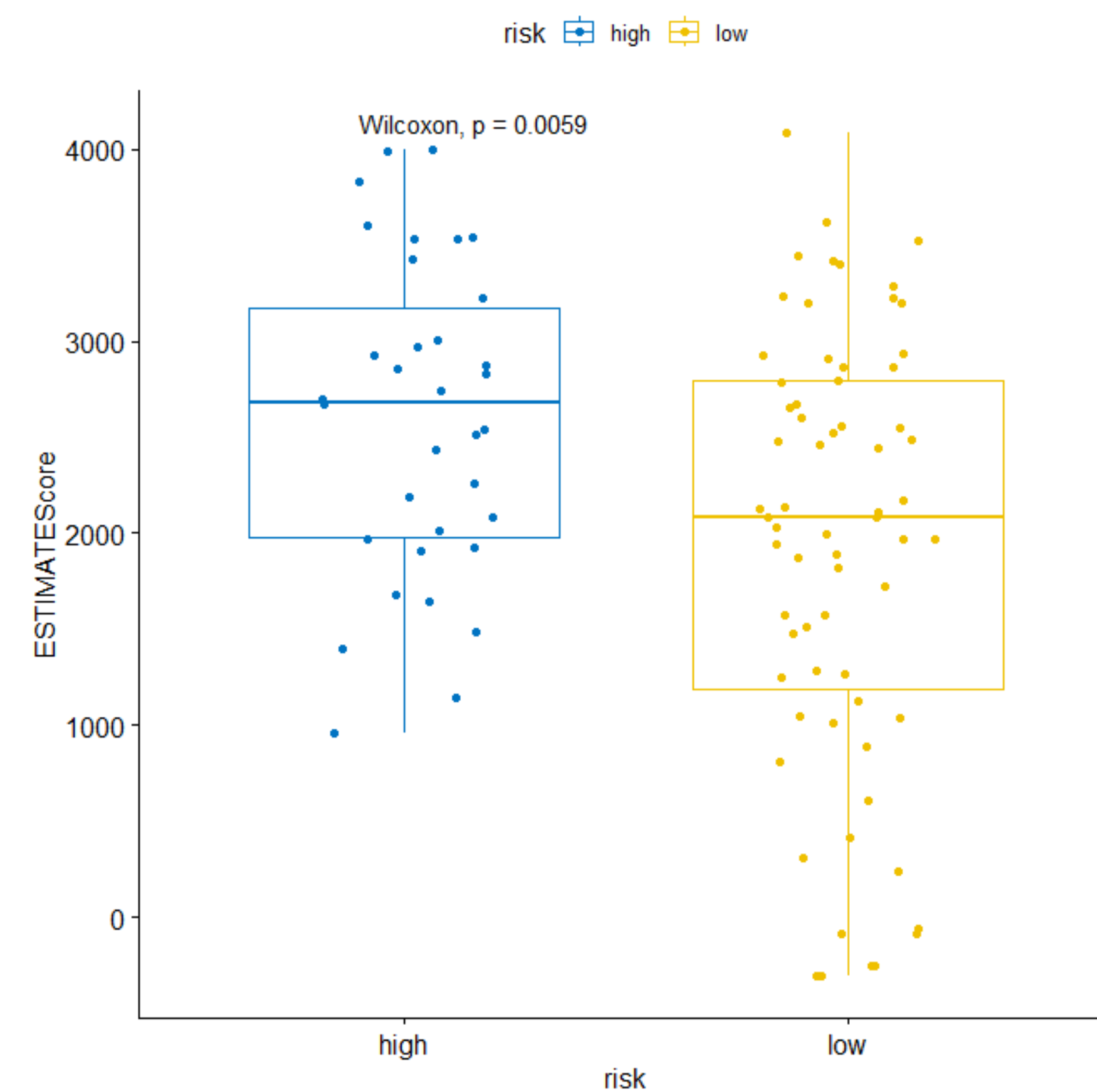

H

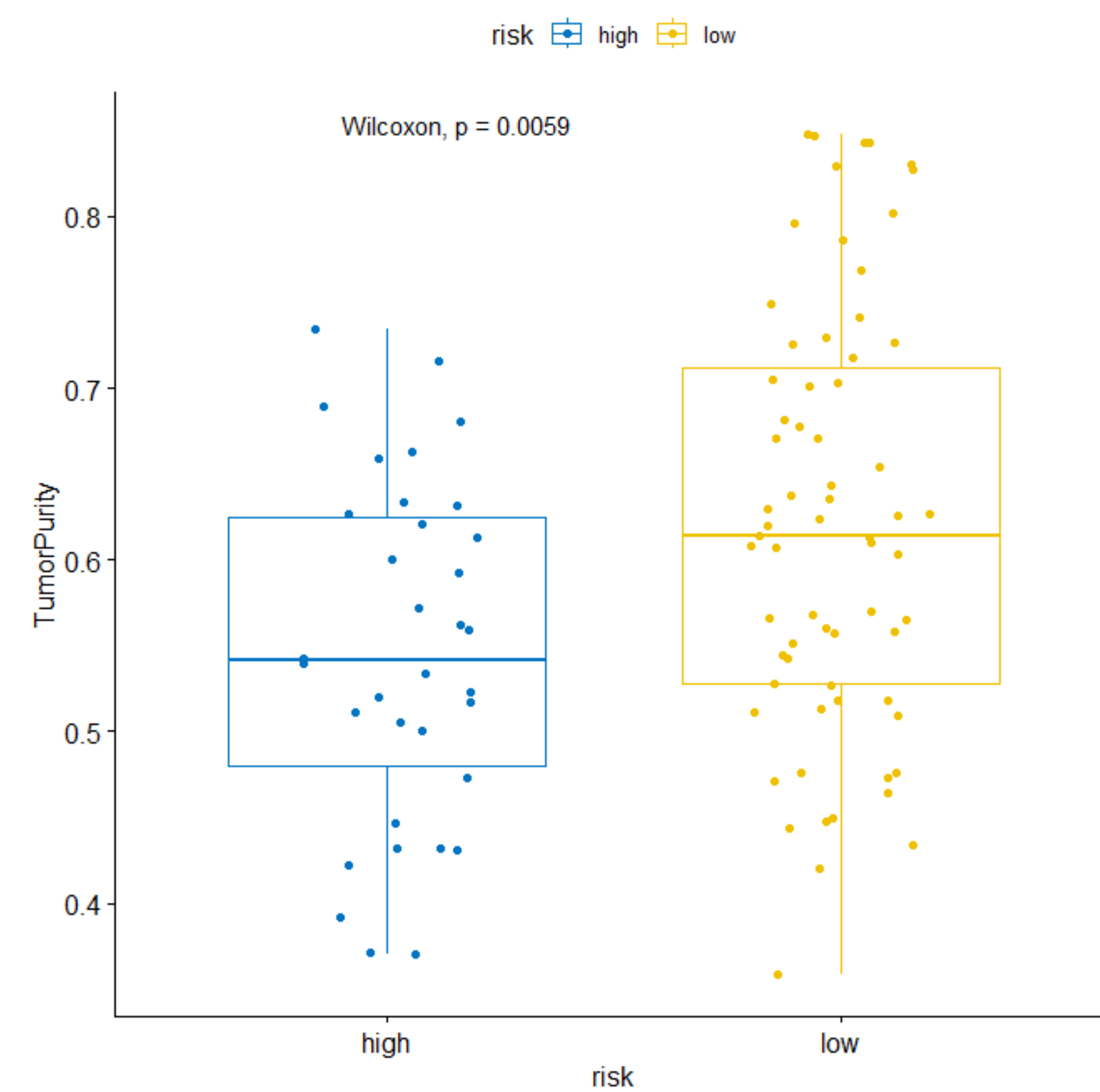

Supplement: Supplementary file 3 — Figure S3: The assessment of TME in female GC patients from external validation cohorts. (A–D) Stromal score, immune score, ESTIMATE score, and tumor purity in GSE15459. (E–H) Stromal score, immune score, ESTIMATE score, and tumor purity in GSE62254. [file CNR2-9-e70469-s003.pdf]
